# Supplementary material for: Transcription-coupled and epigenome-encoded mechanisms direct H3K4 methylation
Source: Nat Commun. 2022 Aug 11;13:4521. doi: 10.1038/s41467-022-32165-8 (PMC9372134; doi:10.1038/s41467-022-32165-8)
Supplement: Supplementary file 1 — Supplementary Information [file 41467_2022_32165_MOESM1_ESM.pdf]

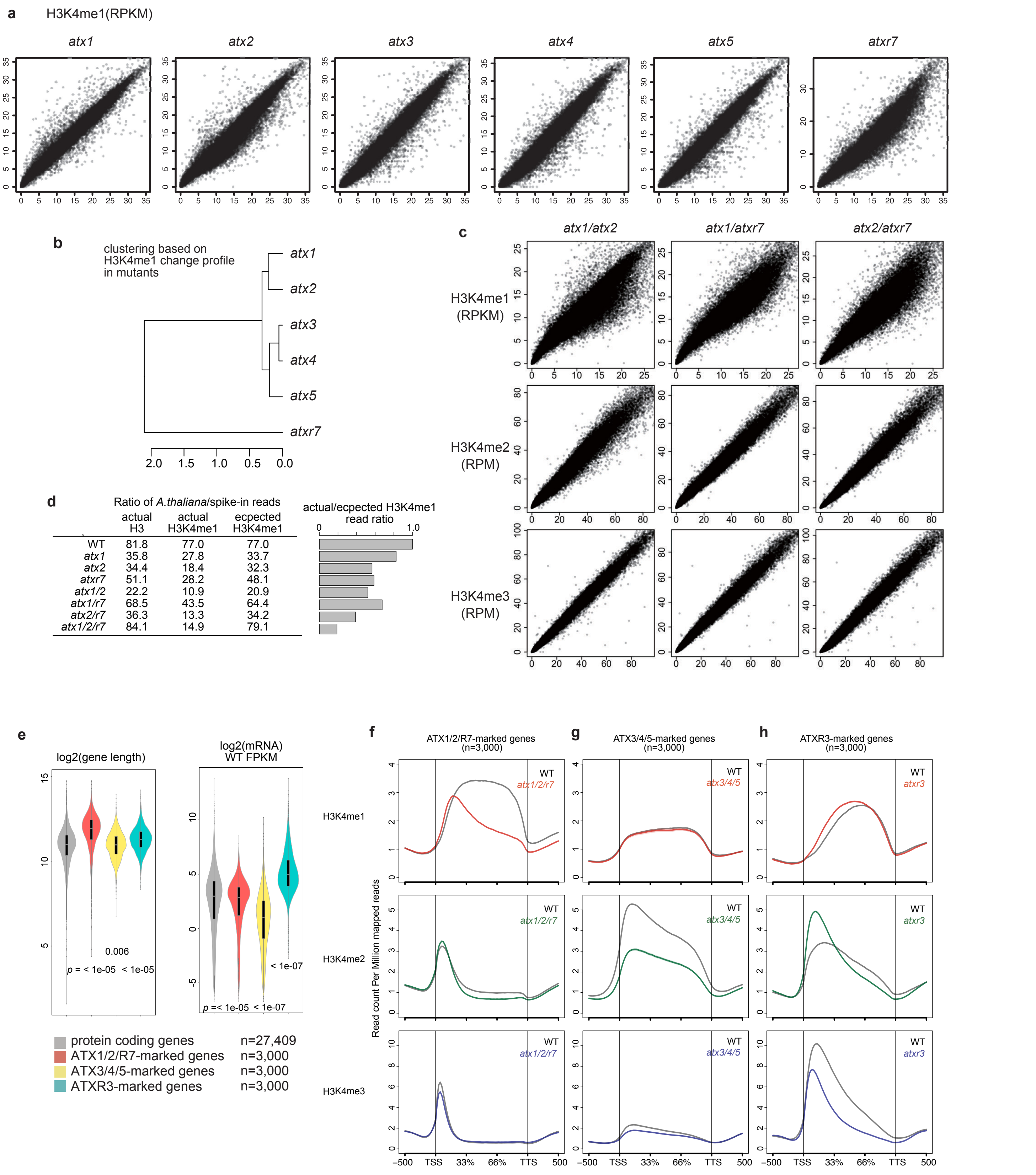

Supplementary Figure 1 Impacts of *atx(r)* mutations on H3K4me

(a) ChIP-seq of H3K4me1 in 6 mutants (y-axis) did not show large change compared to WT (x-axis). Each dot represents one gene and axes are trimmed to 0.98 quantiles. **(b)** *atx1,2,3,4,5* and *atxr7* mutants were clustered based on the H3K4me1 pattern detected in ChIP-seq (see Methods). **(c)** ChIP-seq for H3K4me1, H3K4me2, H3K4me3 in *atx1/2, atx1/atxr7 and atx2/atxr7* double mutants (y-axis) compared to WT (x-axis). For all three mutants, H3K4me1 shows the most prominent reduction. **(d)** Spike-in normalization shows mutations of *atx1*, *atx2,* and *atxr7* synergistically cause reduced H3K4me1. The raw read counts are provided in Supplementary Data 2. **(e)** ATX1/2/R7-, ATX3/4/5-, and ATXR3-marked genes have distinct characteristics in gene length and expression level. The center line of violin plot, median; box limits, upper and lower quartiles; whiskers, 1.5x interquartile range. Numbers within the figure represent p-values calculated with two-sided Dunnett's test comparing against protein coding genes. **(f)-(h)** Metaplots illustrating the averaged distribution of H3K4me1,2,3 over ATX1/2/R7-marked **(f)**, ATX3/4/5-marked **(g)**, and ATXR3-marked genes **(h)**.

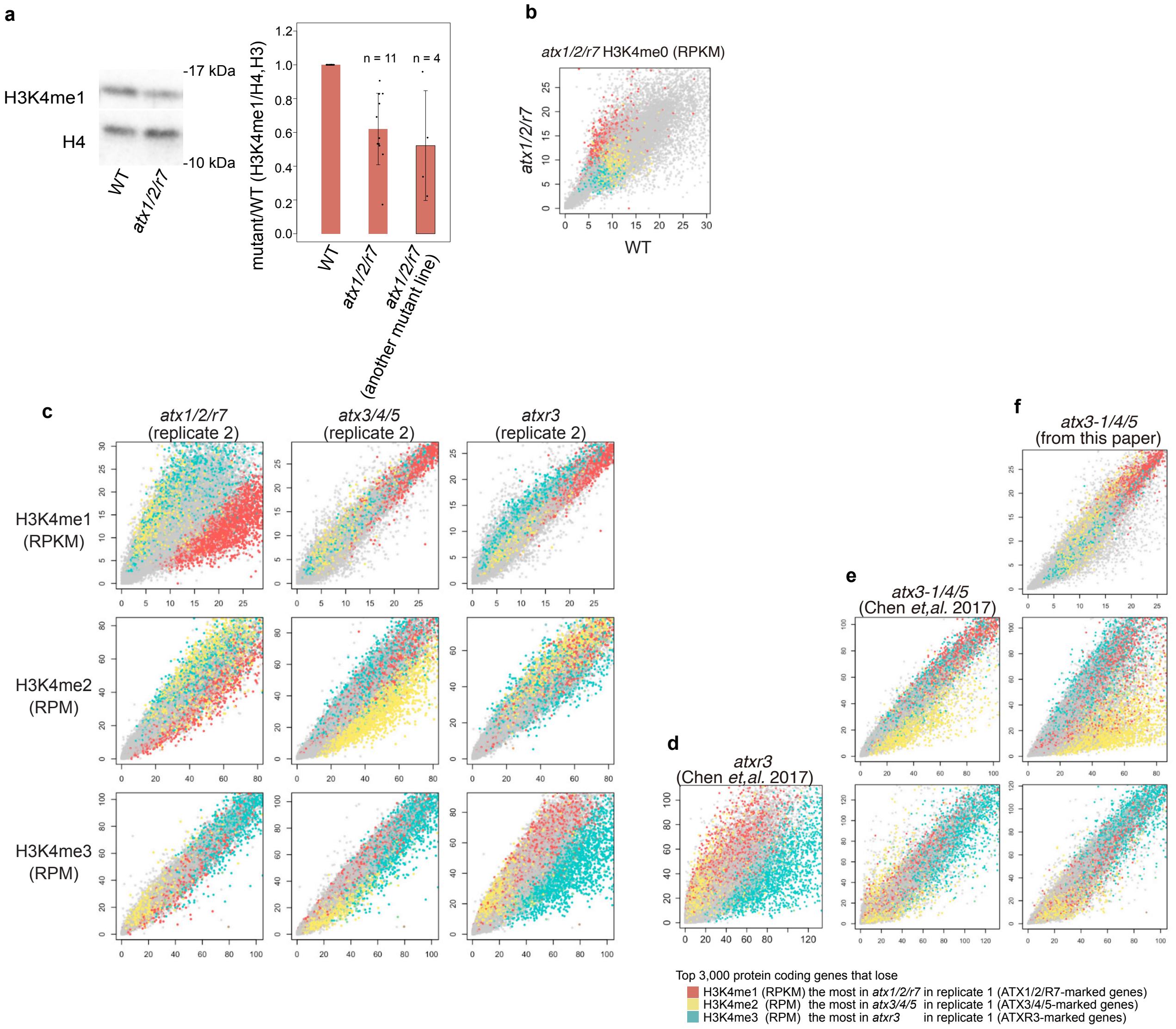

Supplementary Figure 2 Effects on H3K4me in mutants agree with previous reports and replicates.
(a) Western blot for H3K4 methylations on bulk histone extracted from the mutants. Left panel shows example blots for H4 and H3K4me1 obtained from the same membrane. Right panel shows H3K4me1 signals normalized to H3 or H4 histone signals in two lines of *atx1/2/r7* mutants compared to WT. Data are presented as mean±SD and each dot represents data of each replicate. **(b-f)** Mutant (y-axis) ~ WT (x-axis) comparison of H3K4me profiles. In all panels, genes are colored with the same rules defined in Figure. 1d. **(b)** ChIP-seq targeting H3K4me0 shows that in *atx12r7*, hypo-H3K4me1 genes gain H3K4me0, consistent with the view that ATX1,2, and ATXR7 are responsible for H3K4me0-to-H3K4me1 conversion. **(c)** Biological replicates of H3K4me ChIP-seq in *atx1/2/r7, atx3/4/5 and atxr3*. **(d-f)** Previously reported ChIP-seq datasets in *atxr3* (**d**), *atx3-1/4/5* (**e**) mutants, and our datasets on *atx3-1/4/5* (**f**). Our datasets show consistent trends with datasets from ref. 20; in both datasets, *atxr3* mutants lose H3K4me3 in blue-colored genes while *atx3(-1)/4/5* mutants lose H3K4me2 in yellow-colored genes. Axes are trimmed to 0.98 quantiles.

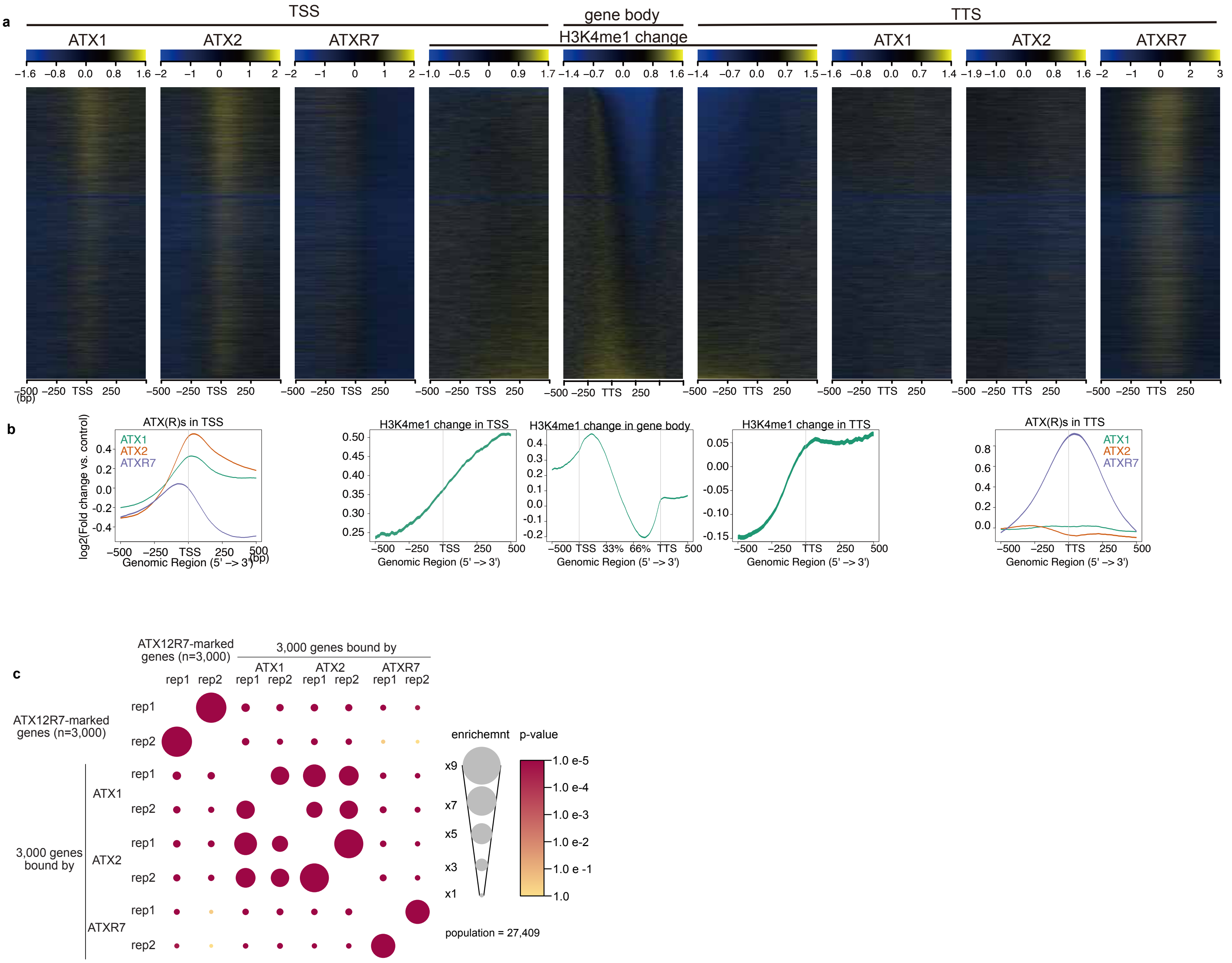

Supplementary Figure 3 ATX1, ATX2, and ATXR7 occupied genes tend to lose H3K4me1 in *atx1/2/r7*.
(a) Heat maps showing H3K4me1 change in *atx1/2/r7* and ATX(R)s distribution. All heat maps are sorted according to the H3K4me1 change in *atx1/2/r7* (the heat map in the middle is the same data as Fig. 1c, shown for reference) so that genes that lost H3K4me1 the most come to the top. **(b)** All genes metaplot corresponding to the panel **(a)** above. **(c)** Overlap between ATX12R7-marked genes and ATX1, ATX2, ATXR7-bound genes. Enrichment is the actual/expected number of overlapped genes, p-values are calculated with hyper-geometric test.

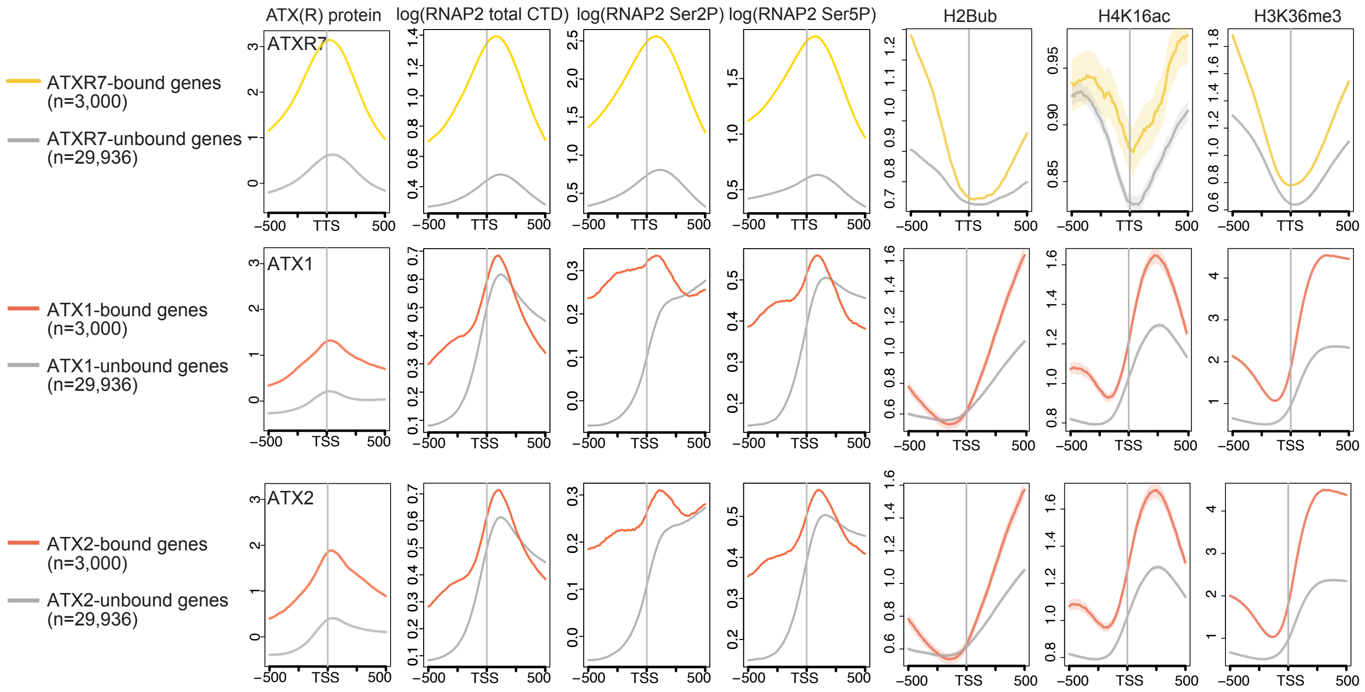

Supplementary Figure 4 Distribution profiles of predictively ‘important’ chromatin modifications for ATX1, ATX2, or ATXR7 localization.
Averaged distribution profiles of ATX(R)s proteins and features that had high importance in the random forest models to predict ATXR7 or ATX1,2-bound genes (see Fig. 3). Profiles around TTS are shown for ATXR7, and profiles around TSS are shown for ATX1 and ATX2.

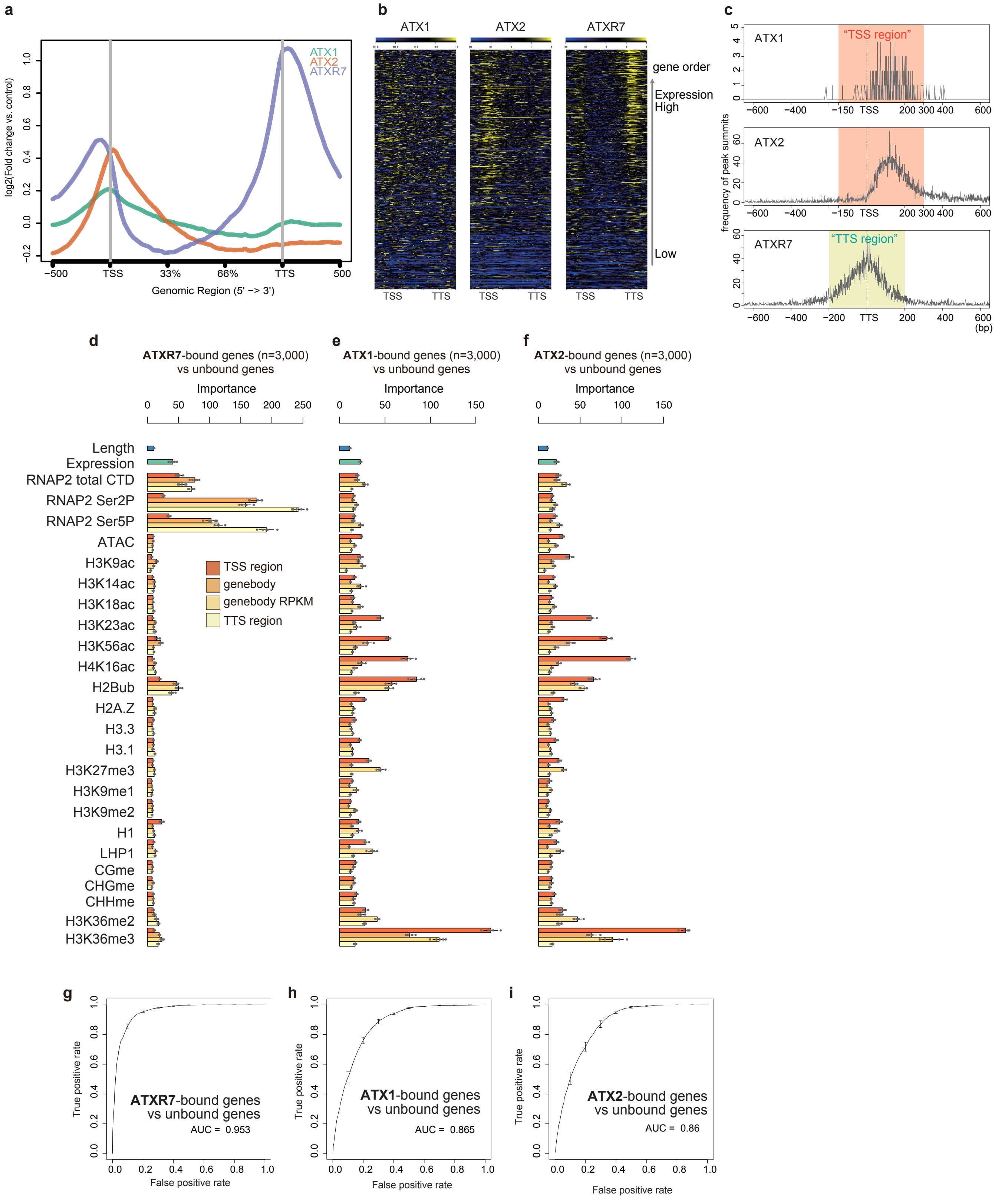

Supplementary Figure 5 Random forest analyses using the biological replicates of ATX(R)s ChIP-seq.
The ChIP-seq data sets of the biological replicates, which were independently grown, were processed in the same manner as in Fig. 2 and Fig. 3. **(a)** Metaplot and **(b)** heat maps illustrating ATX1, ATX2, and ATXR7 distributions in the gene body region corrected with non-transgenic control. The heat maps were sorted so that highly transcribed genes (measured as mRNA-seq in WT) come to the top. **(c)** Position of ATX1 and ATX2 ChIP-seq peaks relative to TSS and ATXR7 peaks relative to TTS (x-axis), visualized as a frequency of peak summits (y-axis). Consistent with the other replicates (Fig. 2), most of ATX1 and ATX2 peaks belong to the 'TSS region', while most of ATXR7 peaks belong to the 'TTS region'. **(d-f)** '**Importance**' of features and **(g-i)** ROC plot of random forest model trained to predict ATX1/2/R7-bound genes. Average and standard deviation of the 5 repeats of training are plotted.

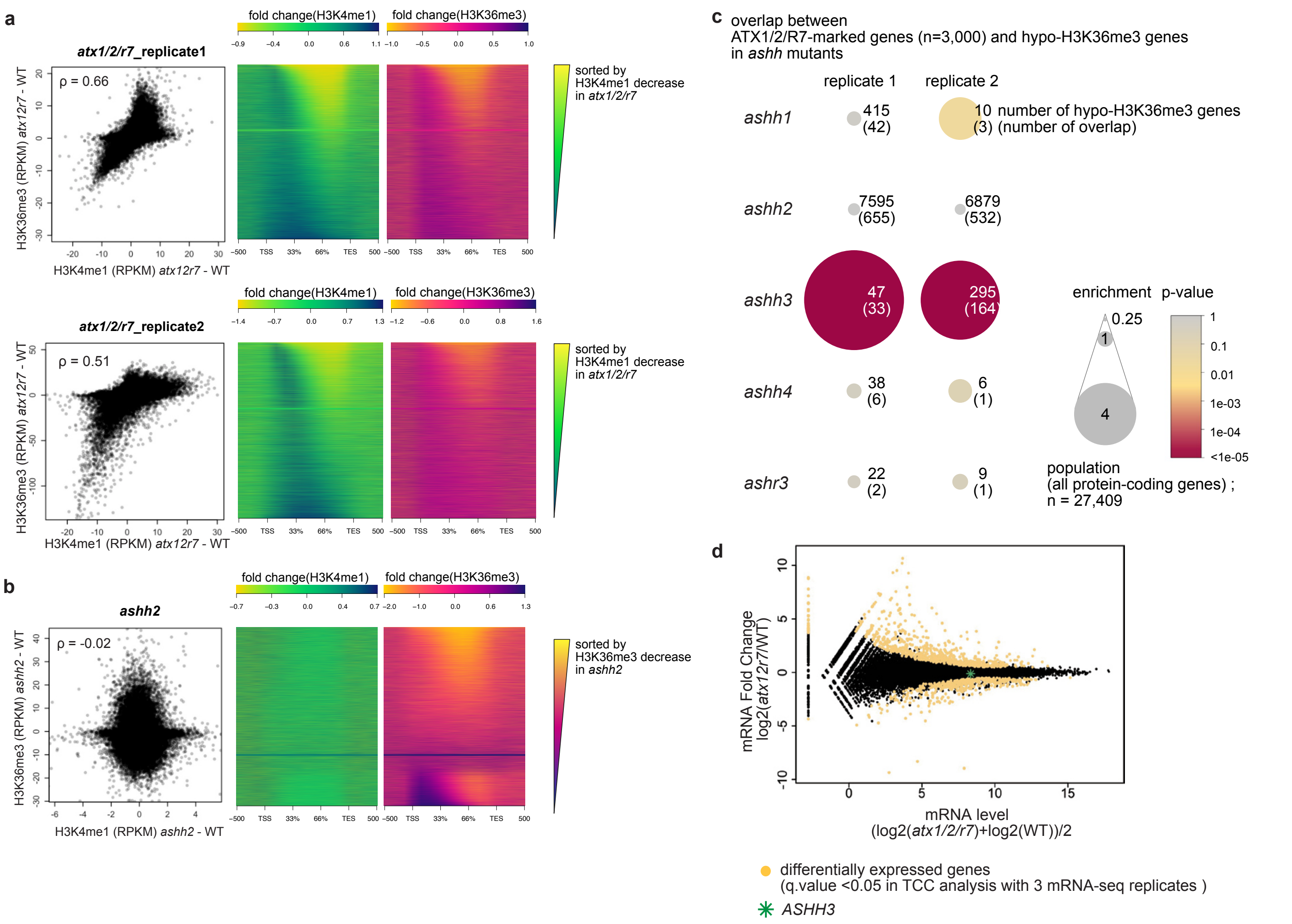

Supplementary Figure 6 H3K4me1 may promote H3K36me3 via ASHH3.
(a) In *atx1/2/r7*, hypo-H3K4me1 correlates with H3K36me3 loss, while change of H3K36me3 levels in *ashh2* **(b)** does not correlate with change of H3K4me1. Each dot in the scatter plot represents one protein coding gene and axes are trimmed to 0.0005 and 0.9995 quantiles. Heat maps in **(a)** are sorted according to the H3K4me1 change in *atx1/2/r7* (the heat map for H3K4me1 of replicate 1 is the same data as Fig. 1c, shown for reference) so that genes that lost H3K4me1 the most come to the top, while heat maps in **(b)** are sorted according to the H3K36me3 change in *ashh2*. ρ is the Spearman's correlation coefficient. **(c)** In search for a methyltransferase that catalyzes H3K36me3 in response to ATX(R)s-catalyzed H3K4me1, we analyzed H3K36me3 change by ChIP-seq in mutants of ASHH2, known major H3K36me3 methyltransferase [31,32] and ASHH1, ASHH3, ASHH4, and ASHR3, whose catalytic domain SET are similar with ASHH2 [17,18]. Overlap between ATX1/2/R7-marked genes and hypo-H3K36me3 genes detected by MACS2 in each *ashh* mutants are shown as bubble plots. The size of a bubble shows enrichment value against all protein-coding genes (n = 27,409) and the p-value represented by color is based on unadjusted one-sided hypergeometric test. *ashh3* lose H3K36me3 at ATX1/2/R7-marked locus, suggesting that ASHH3 may functions downstream of ATX1/2/R7-marked H3K4me1 to mediate H3K36me3. **(d)** mRNA expression of *ASHH3* was not altered in *atx1/2/r7*, precluding the possibility that H3K36me3 change in *atx1/2/r7* was due to reduced expression of *ASHH3*.

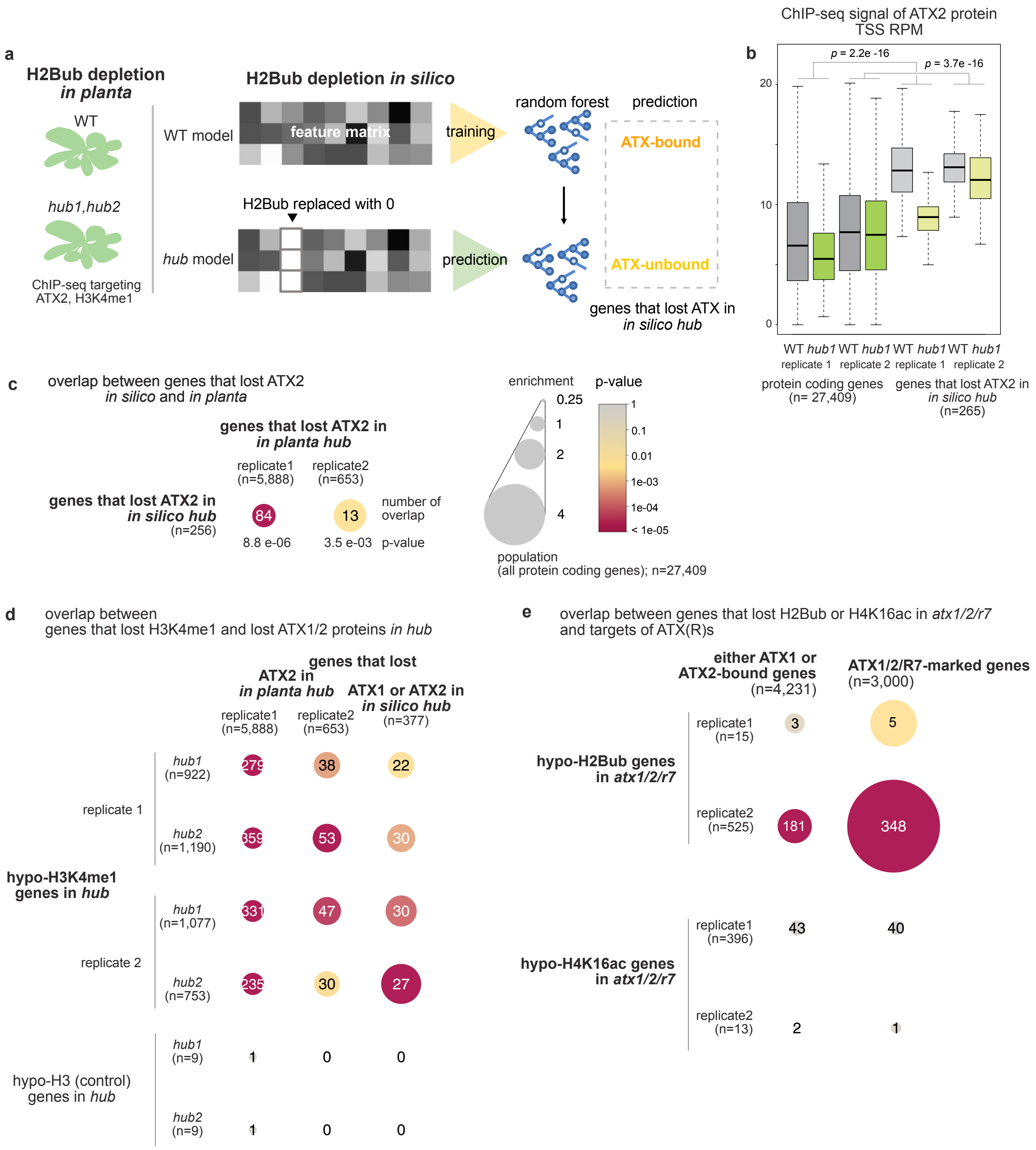

Supplementary Figure 7 Loss of H2Bub results in loss of ATX2 localization and decreased H3K4me1. **(a)** Overview of the analysis. To test if H2Bub promotes presence of ATX, ChIP-seq analysis of ATX2 localization was performed in *hub2* and WT (control) background in two replicates. Mutation in either *HUB1 or HUB2* is known to depletes H2Bub to undetectable levels[42,102–104]. ATX localization in the absence of H2Bub was simulated by letting the trained random forest model (WT model) predict ATX-bound genes using a feature table in which H2Bub are replaced with 0 (*hub* model). 'Genes that lose ATX in *in silico hub*' are the genes that are predicted to be ATX-bound in the WT model and unbound in the *hub* model. **(b)** *In planta* ChIP-seq signal over the TSS region of 'genes that lose ATX in *in silico hub*' is largely reduced in *hub2* compared to WT. The ratio of wild-type to hub ATX2 signal was significantly different for all genes vs 'Genes that lose ATX in *in silico hub*', as indicated by the p-value of Wilcoxon's rank sum test. Center line of boxplot, median; box limits, upper and lower quartiles; whiskers, 1.5x interquartile range. **(c)** Genes that are detected to lose ATX2 *in planta* matches *in silico* prediction. Genes that correspond to hypo-ATX regions, which are detected by comparing ATX2 ChIP-seq profile in *hub2* mutant and WT background using peak caller MACS2, significantly overlap with genes predicted to lose ATX in the *in silico hub*. Numbers within the circles are the number of overlaps and the p-value represented by color is based on unadjusted one-sided hypergeometric test. Size and color scales are the same in **(c)**-**(e)**. **(d)** To further test if H2Bub promotes ATX1,2, thereby H3K4me1, H3K4me1 ChIP-seq was performed in *hub1*, *hub2* and WT. Genes hypo-H3K4 monomethylated in the *hub* significantly overlap with genes that lose ATX2 in the *in planta hub2* (2 replicates) and genes that lose ATX1 or ATX2 in the *in silico hub*. **(e)** To test if H3K4me1 promotes H2Bub or H4K16ac, ChIP-seq targeting H2Bub and H4K16ac was performed in *atx1/2/r7* and WT. Genes that are hypo-H2B ubiquitinated in *atx1/2/r7* significantly overlap with hypo-H3K4me1 genes (= ATX1/2/R7-marked genes), while genes detected to be hypo-H4K16 acetylated did not overlap with ATX1/2/R7-marked genes nor with ATX1,2 localization, suggesting that H2Bub but not H4K16ac is regulated downstream of H3K4me1.

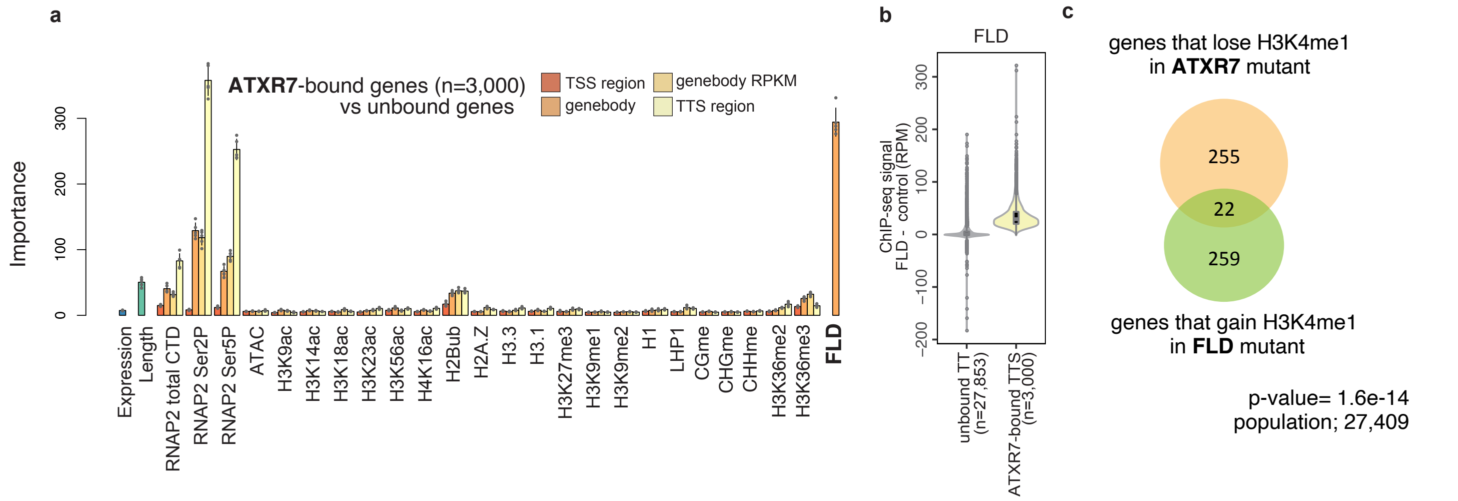

Supplementary Figure 8 ATXR7 and FLD share target genes.
(a) ATXR7 and FLD colocalize. When the Random Forest model is trained to predict ATXR7-bound and -unbound genes in the same manner as Fig. 3a except for the inclusion of the amount of FLD protein in the TTS region showed high importance. Error bars represent the standard deviation of the 5 repeats of training. **(b)** ATXR7-bound TTS regions are highly enriched in FLD. **(c)** There is a significant overlap between genes that lose H3K4me1 in the *atxr7* mutant (RPKM WT-*atxr7* > 6.5) and genes that gain H3K4me1 in the *fld* mutant. p-value is calculated with a hypergeometric test. The center point of violin plot, median; box limits, upper and lower quartiles; whiskers, 1.5x interquartile range.

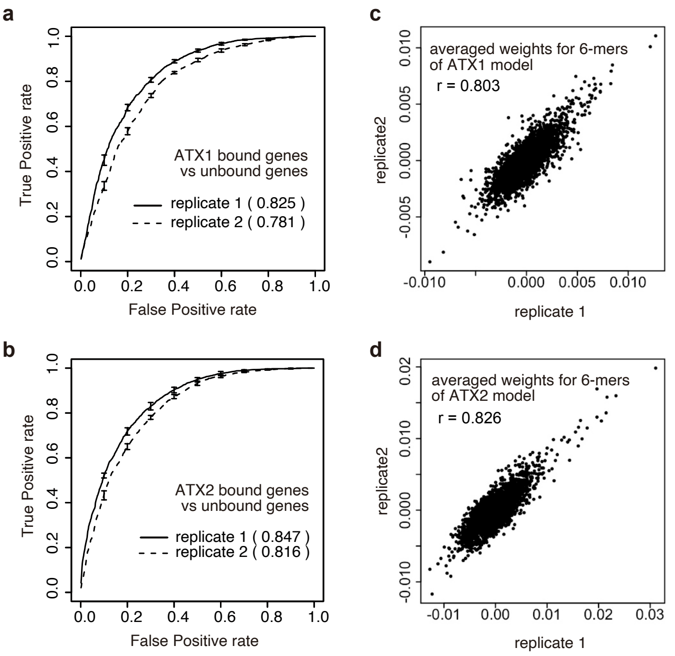

Supplementary Figure 9 Biological replicates confirmed that DNA motifs also rule ATX1 and ATX2 localization. The ChIP-seq data sets of the biological replicates (Supplementary Fig. 5) were analyzed in the same manner as Fig. 4. Biological replicates also achieved accurate prediction for both **(a)** ATX1 and **(b)** ATX2 as demonstrated by ROC plots. Error bars represent the standard deviation of the 5 repeats of training. **(c,d)** The learned trends of weights highly correlated between the replicates. r represents Pearson’s correlation coefficient.

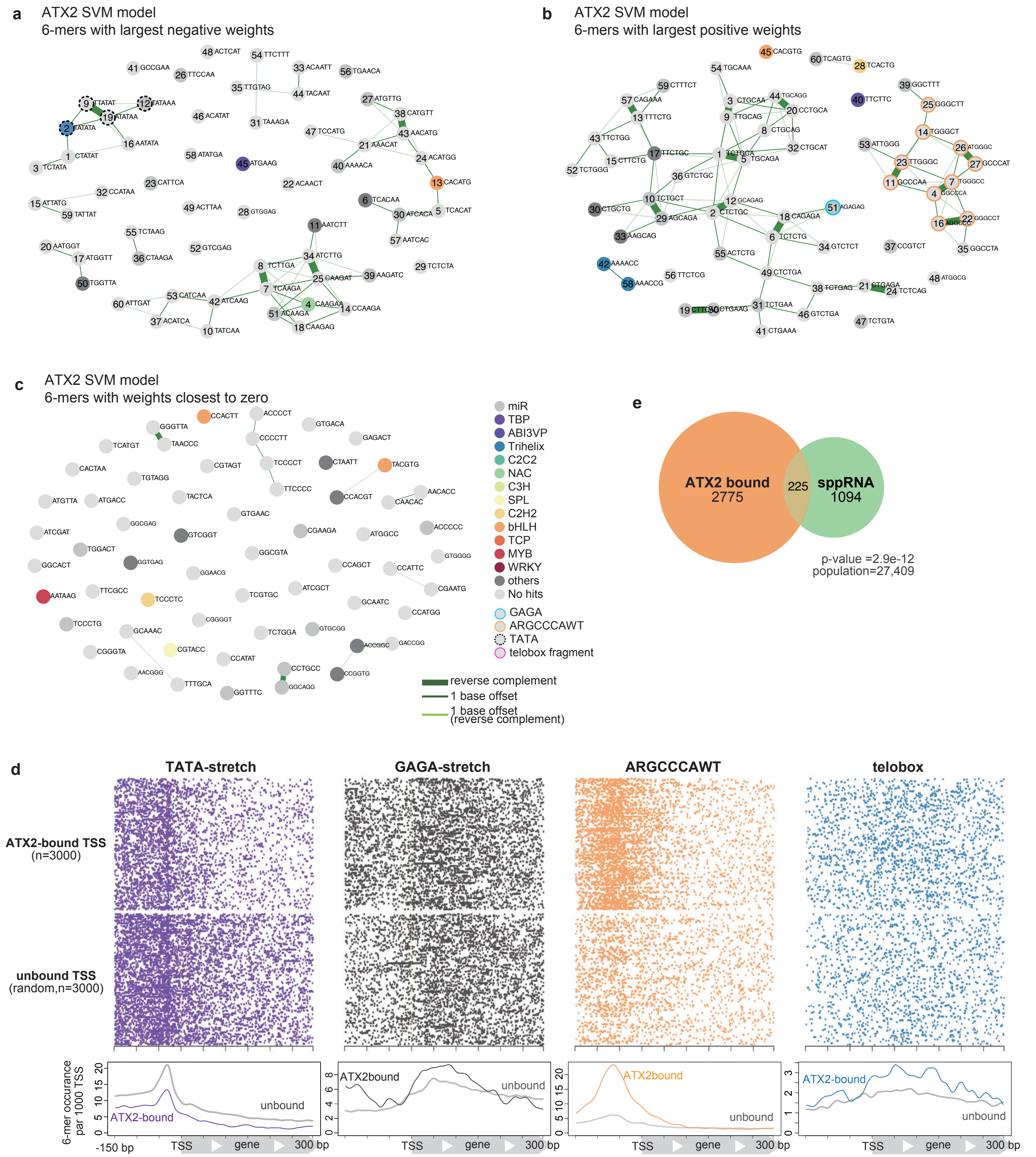

Supplementary Figure 10 Annotations of predictive and non-predictive 6-mers in the ATX2 model. Predictive and non-predictive 6-mers in the ATX2 models were selected and analyzed in the same manner as in ATX1 models (Fig. 4). **(a-c)** Clustering and annotation of predictive and non-predictive 6-mers. Each circle represents the top sixty 6-mers with negative (**a**), positive (**b**), or nearly zero (**c**) SVM weights in the ATX2 model. **(d)** Positional distributions of highly weighted motifs in the TSS region. **(e)** ATX2-bound TSS significantly overlaps with sppRNA-harboring TSS detected in the *hen2-2* background. The significance of the overlap was tested using a hypergeometric test.

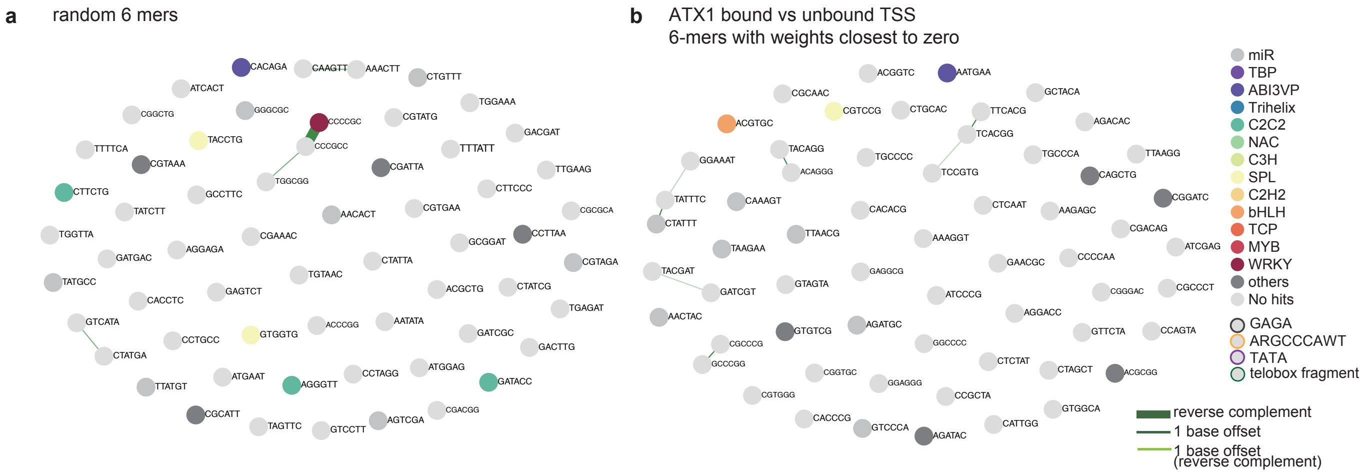

Supplementary Figure 11 Non-predictive 6-mers.
(a,b) Clustering and annotation of non-predictive sixty 6-mers. Random (**a**) and near-zero-weighted (**b**) sixty 6-mers in the ATX1 model.

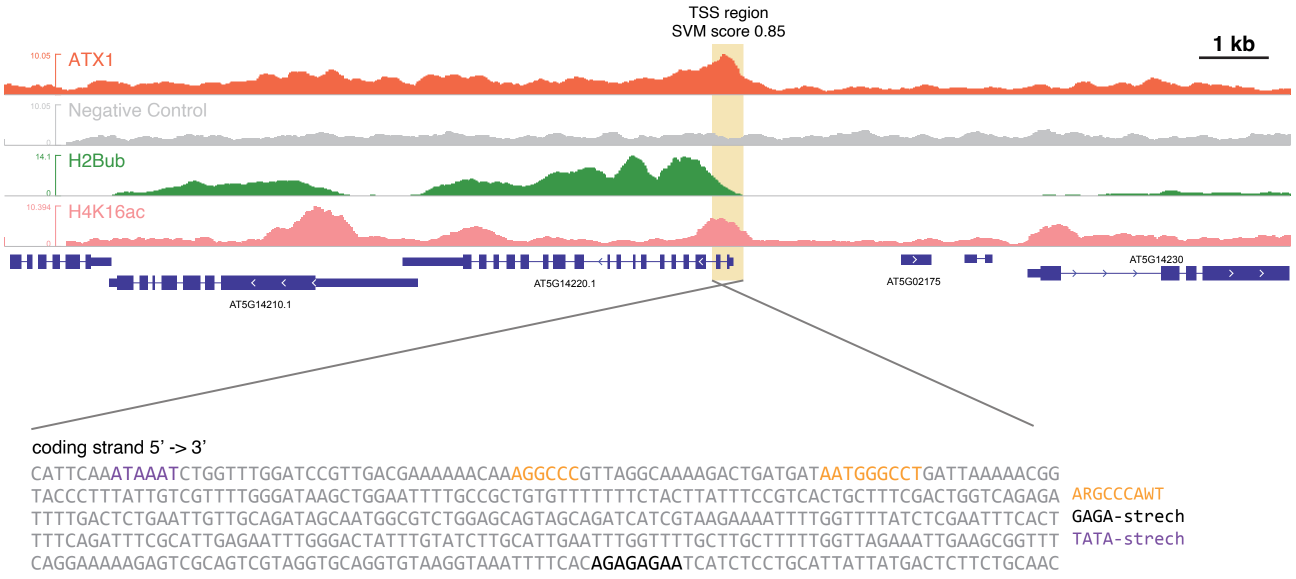

Supplementary Figure 12 An example browser view of ATX1-bound and unbound genes.
Examples of the ATX1-bound and unbound genes. ChIP-seq read coverage tracks of ATX1, negative control (non-transgenic), H2Bub, and H4K16c are shown. AT5G14220 is one of the ‘ATX1-bound genes’. Its ‘TSS region’, highlighted with yellow shade, has higher levels of H2Bub and H4K16ac compared to the other TSS regions in the view. The SVM score of this TSS region in the full model is 0.85 (ranges from 0 to 1. The higher the value is, the more likely it to be ATX1-bound, based on DNA sequence). In the DNA sequence of the TSS region, positively weighted ARGCCCAWT and GAGA-stretch are present, as well as negatively weighted TATA-stretch.

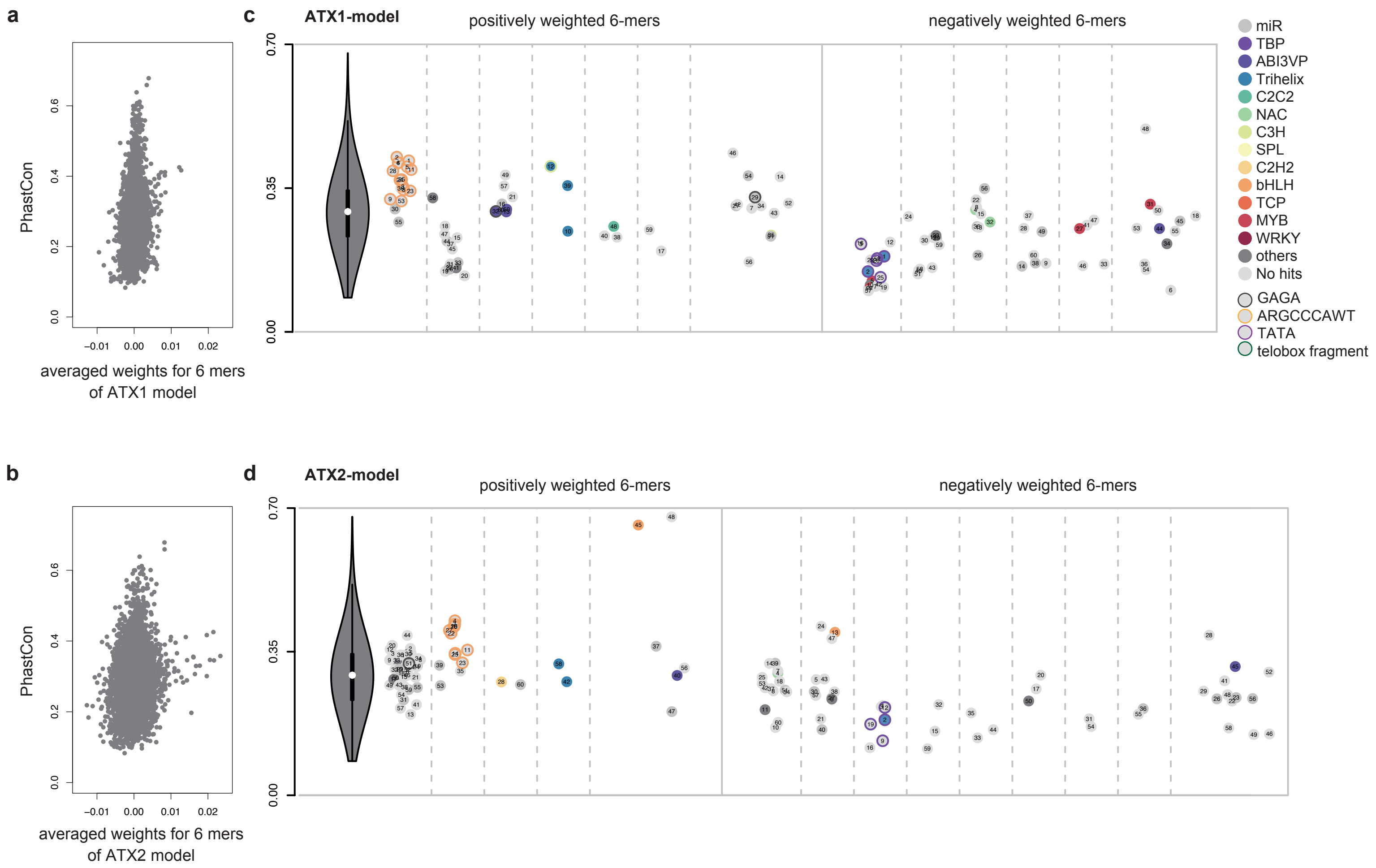

Supplementary Figure 13 Conservation of the predictive motifs in angiosperm genomes.
The conservation score PhastCon[99] score calculated by ref. 101 based on genome sequence alignment of 63 angiosperm species. Higher PhastCons score reflects higher conservation within the TSS region. **(a,b)** SVM weights and PhastCons lack overall correlation **(c,d)** RGCCCAW motif is under evolutionary constraint. Leftmost violin plots represent PhastCon distribution of all 6-mers (n= 4^6 = 4096) in the TSS region. The center point of violin plot, median; box limits, upper and lower quartiles; whiskers, 1.5x interquartile range. Each circle represents highly weighted 6-mers with the same color and numbers as Fig. 4 and Supplementary Fig. 10. 6-mers connected with lines in Fig. 4 and Supplementary Fig. 10 are clustered together.

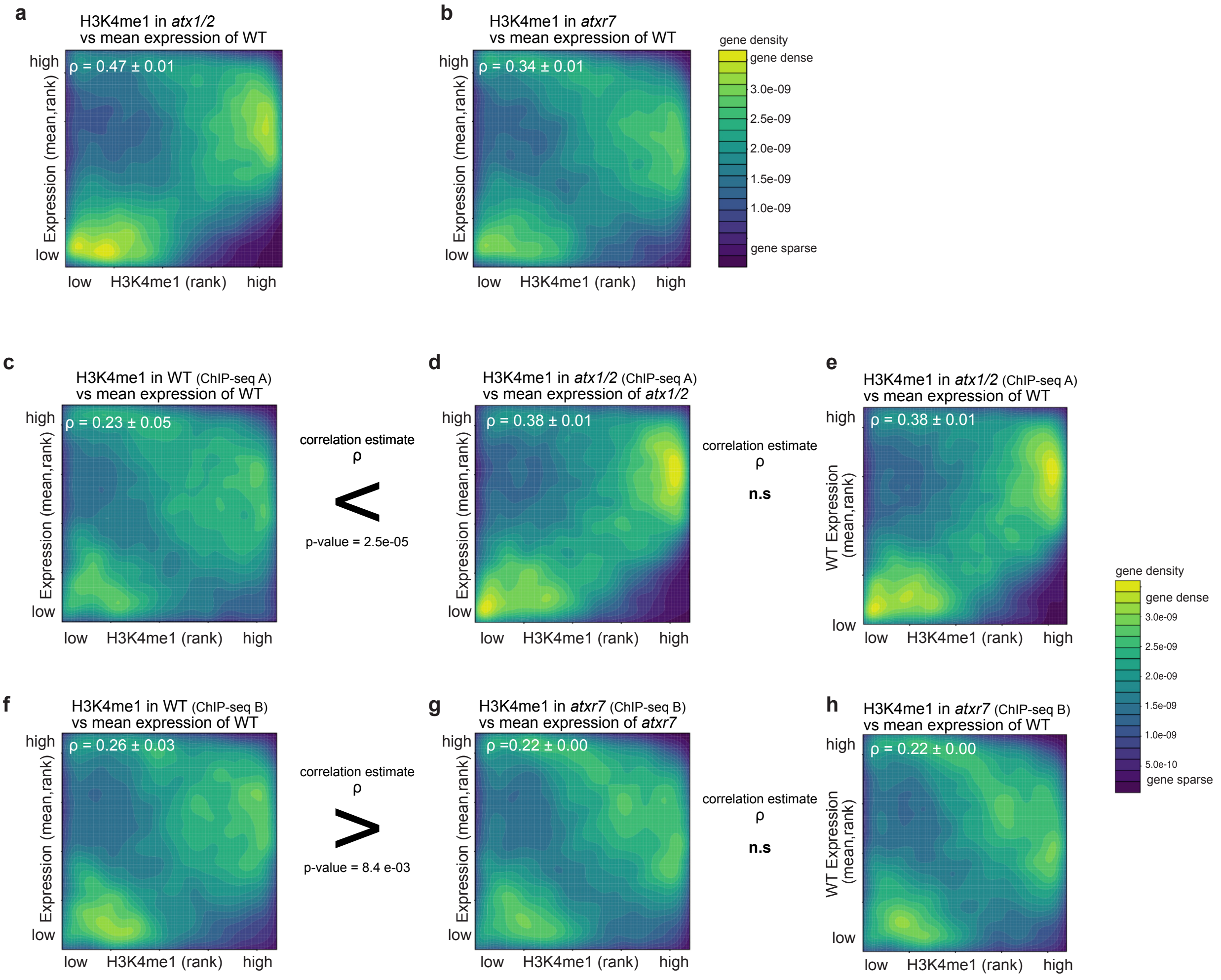

Supplementary Figure 14 Change in H3K4me1-transcription correlation is due to change in H3K4me1, not in transcription. (a,b) Correlation between H3K4me1 and expression is shown in the same way as Fig. 5, except that in this figure the expression (mRNA-seq) data are of WT. The *atx1/2* mutant has a higher, while *atxr7* has a lower correlation than WT, demonstrating that the change in H3K4me1 rather than transcription causes the change in the correlation. ρ= Spearman's correlation coefficient. The color scale is the same as Fig. 5. **(c-h)** Another ChIP-seq datasets replicated the trend. ChIP-seq data used for **(c-e)** *atx1/2* -WT comparison is the data sets for double mutants shown in Supplementary Fig. 1c (ChIP-seq A), and for **(f-h)** *atxr7*-WT comparison is the datasets shown in Supplementary Fig. 1a (ChIP-seq B). P-values indicate ρ are significantly different between genotypes (Welch's two sample t-test).

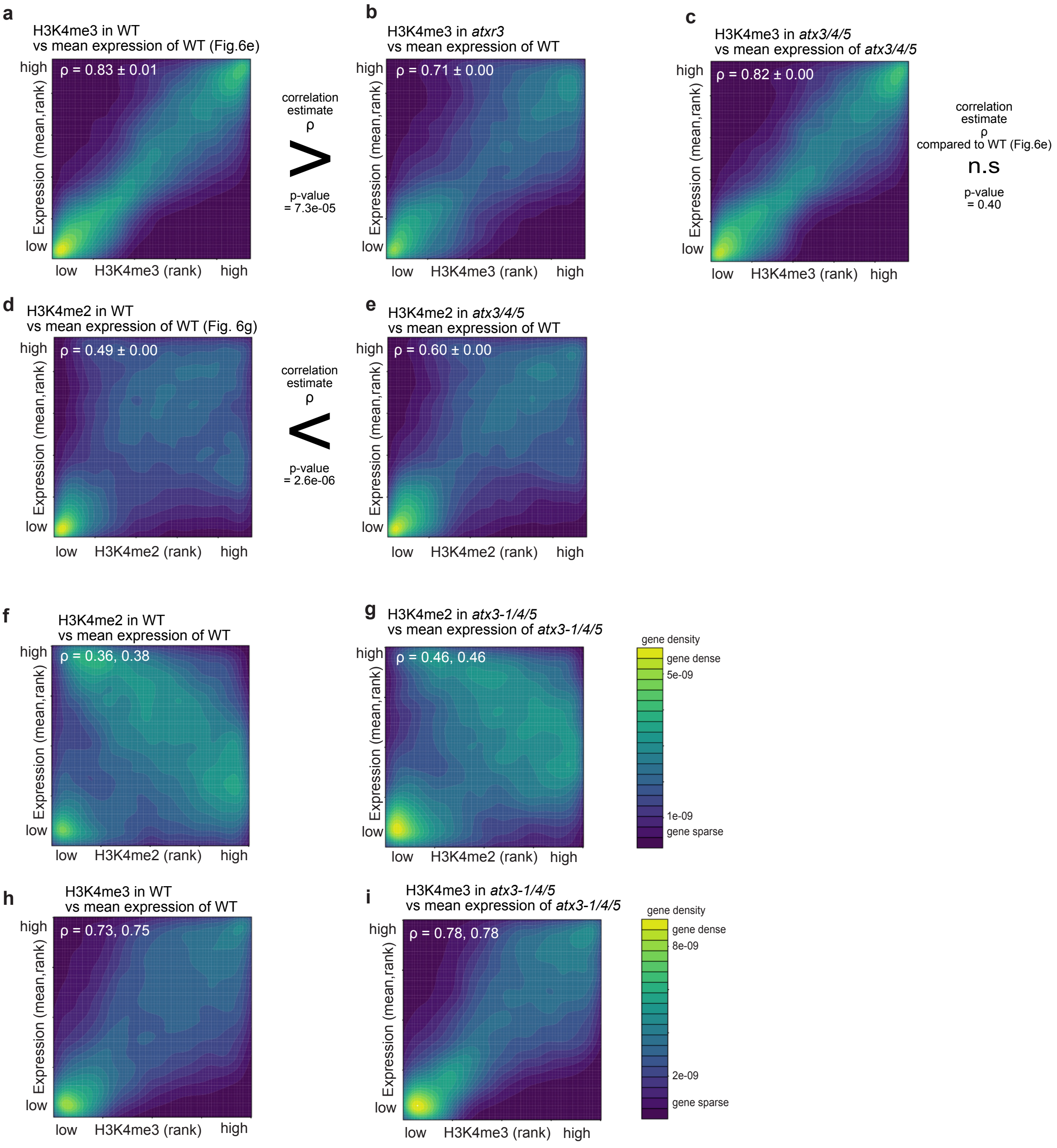

Supplementary Figure 15 Correlational trend between H3K4me and transcription of *atxr3* and *atx3/4/5*.
(a) Identical to Fig. 6e and shown for reference. **(b)** Correlation between H3K4me3 level in *atxr3* and expression level in WT. Compared to WT-WT comparison in **(a)**, the correlation is diminished and almost as weak as a mutant-mutant comparison in Fig. 6f, demonstrating that the change in H3K4me3 rather than transcription causes the change in the correlation. **(c)** *atx3/4/5* mutant does not show a large correlational change between H3K4me3 and transcription. **(a-c)** and Fig. 6e,f are shown in the same color scale. **(d)** Identical to Fig. 6g. **(e)** Correlation between H3K4me2 level in *atx3/4/5* and expression level in WT. Compared to a WT-WT comparison in **(d)**, the correlation is stronger and almost as strong as a mutant-mutant comparison in Fig. 6h, demonstrating that the change in H3K4me2 rather than transcription causes the change in the correlation. **(f-i)** Change in the correlation between H3K4me2 **(f, g)** or H3K4me3 **(h, i)** and transcription in the *atx3-1/4/5* mutant, from the datasets reported by Chen et al., 2017 (ref. 20). In the *atx3-1/4/5* mutant, which has stronger effects on H3K4me2 and H3K4me3 compared with *atx3/4/5* (Fig. 1e, Supplementary Fig. 2), there is a stronger correlation compared to WT not only in transcription-H3K4me2 but also in transcription-H3K4me3. P values indicate ρ are significantly different between genotypes (Welch's two sample t-test).

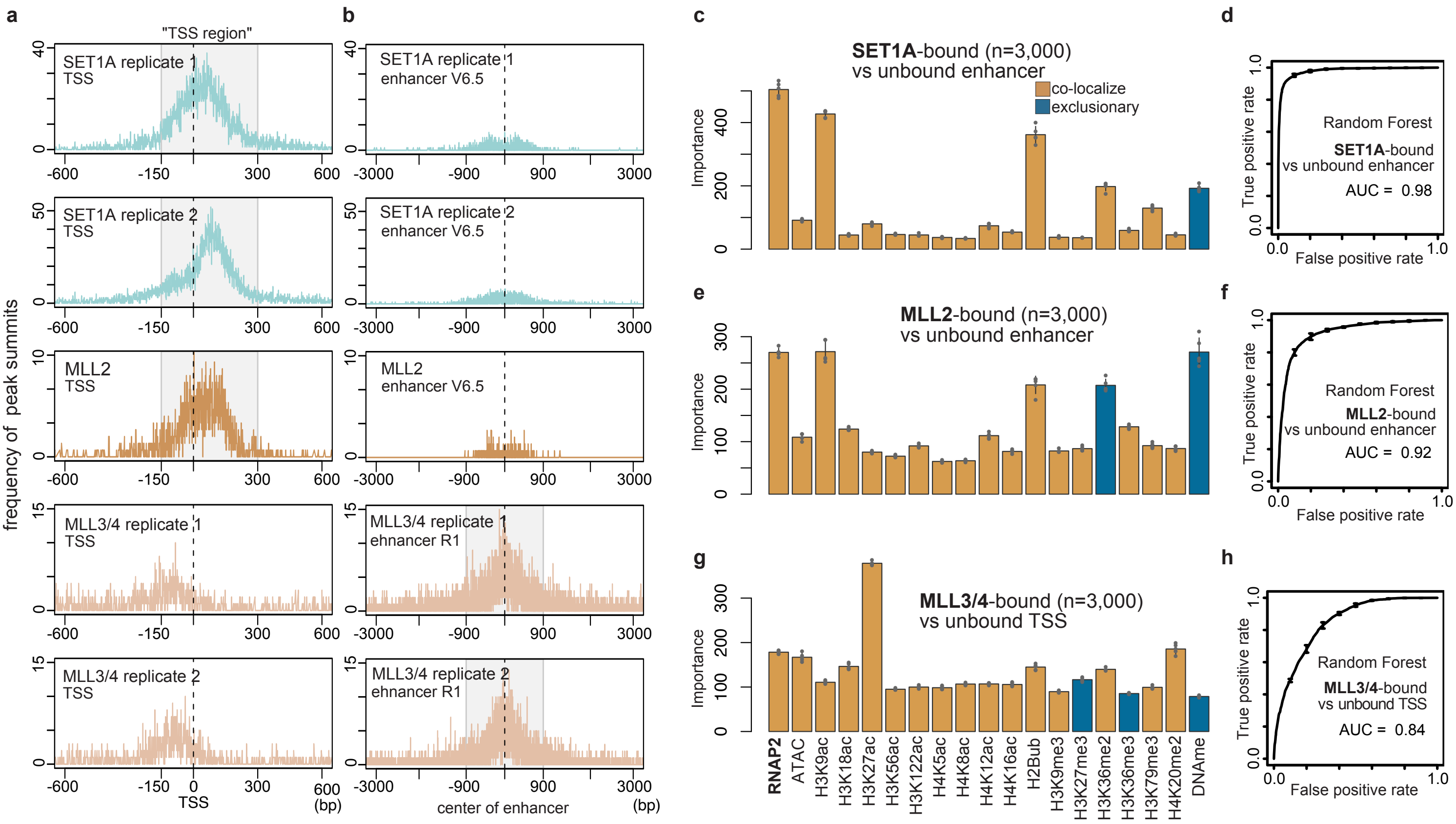

Supplementary Figure 16 Localization of mammalian H3K4 methyltransferases in TSSs and enhancers. **(a,b)** Position of mammalian H3K4 methyltransferases' peaks relative to TSS (**a**) and enhancer (**b**), visualized as a frequency of ChIP-seq peak summits. x-axis, distance from TSS (**a**) or center of enhancer (**b**); y-axis, number of peak summits. Most of SET1A and MLL2's peaks belong to the region spanning from 150 bp upstream to 300 bp downstream of TSS, where we hereafter refer to as the 'TSS region'. Most of the MLL3/4 peaks belong to 1800 bp around the center of the enhancer, where we hereafter refer to as 'enhancer'. **(c,e,g)** 'Importance' of features derived from the Random Forest models trained to predict enhancer occupied by SET1A (**c**) and MLL2 (**e**), or to predict TSS region occupied by MLL3/4 (**g**). Graphs are shown like Fig. 7b,e,h. **(d,f,h)** ROC plot showing the prediction accuracy of the random forest models corresponding to (**c**,**e**,**g**). All ROC and AUC are calculated with test data (25% of the original data). Average and standard deviation of the 5 repeats of training are plotted.

```
ATX1      K R L A F - - - - - G K S G I H G F G I F A K L P - - H R A G D M M I E Y T G E
ATX2      K R L A F - - - - - G K S G I H G F G I F A K L P - - H R A G D M V I E Y T G E
ATX3      F R V C F - - - - - G K S G I H G W G L F A R K S - - I Q E G E M I I E Y R G V
ATX4      D R V C F - - - - - G R S G I H G W G L F A R R N - - I Q E G E M V L E Y R G E
ATX5      E R V C F - - - - - G R S G I H G W G L F A R R N - - I Q E G E M V L E Y R G E
ATXR7     K H L R F - - - - - Q Q S K I H D W G L V A L E P - - I E A E D F V I E Y V G E
ATXR3     K E I E S R S D D K Y V S Y R K G L G V V C N K E G G F G E E D F V V E F L G E
hMLL1     E A V G V - - - - - Y R S P I H G R G L F C K R N - - I D A G E M V I E Y A G N
hMLL2     E A V G V - - - - - Y R S A I H G R G L F C K R N - - I D A G E M V I E Y S G I
hMLL3     S N V Y L - - - - - A R S R I Q G L G L Y A A R D - - I E K H T M V I E Y I G T
hMLL4     N N V Y L - - - - - A R S R I Q G L G L Y A A K D - - L E K H T M V I E Y I G T
hSET1A    K K L R F - - - - - G R S R I H E W G L F A M E P - - I A A D E M V I E Y V G Q
hSET1B    K K L K F - - - - - C K S H I H D W G L F A M E P - - I A A D E M V I E Y V G Q
yeastSET1 K P V M F - - - - - A R S A I H N W G L Y A L D S - - I A A K E M I I E Y V G E
```

```
ATX1      L V - - - - - - - - - R P S I A D K R E Q L I Y N S M V G A G - T Y M F R I D D E
ATX2      L V - - - - - - - - - R P P I A D K R E H L I Y N S M V G A G - T Y M F R I D N E
ATX3      K V - - - - - - - - - R R S V A D L R E A N - Y R S - Q G K D - C Y L F K I S E E
ATX4      Q V - - - - - - - - - R G S I A D L R E A R - Y R R - V G K D - C Y L F K I S E E
ATX5      Q V - - - - - - - - - R G I I A D L R E A R - Y R R - E G K D - C Y L F K I S E E
ATXR7     L I - - - - - - - - - R S S I S E I R E R Q - Y E K - M G I G S S Y L F R L D D G
ATXR3     V Y P V W K W F E K Q D G I R S L Q E N K - T D P A P E F Y N I Y L E R P K G D
hMLL1     V I - - - - - - - - - R S I Q T D K R E K Y - Y D S - K G I G - C Y M F R I D D S
hMLL2     V I - - - - - - - - - R S V L T D K R E K F - Y D G - K G I G - C Y M F R M D D F
hMLL3     I I - - - - - - - - - R N E V A N R K E K L - Y E S - Q N R G - V Y M F R M D N D
hMLL4     I I - - - - - - - - - R N E V A N R R E K I - Y E E - Q N R G - I Y M F R I N N E
hSET1A    N I - - - - - - - - - R Q M V A D M R E K R - Y V Q - E G I G S S Y L F R V D H D
hSET1B    N I - - - - - - - - - R Q V I A D M R E K R - Y E D - E G I G S S Y M F R V D H D
yeastSET1 R I - - - - - - - - - R Q P V A E M R E K R - Y L K - N G I G S S Y L F R V D E N
```

```
ATX1      - - - - - - R V I D A T R T G S I A H L I N H S C V P N C Y S R V I T V - N G D
ATX2      - - - - - - R V I D A T R T G S I A H L I N H S C E P N C Y S R V I S V - N G D
ATX3      - - - - - - I V I D A T D S G N I A R L I N H S C M P N C Y A R I V S M G D G E
ATX4      - - - - - - V V V D A T D K G N I A R L I N H S C T P N C Y A R I M S V G D E E
ATX5      - - - - - - V V V D A T E K G N I A R L I N H S C M P N C Y A R I M S V G D D E
ATXR7     - - - - - - Y V L D A T K R G G I A R F I N H S C E P N C Y T K I I S V - E G K
ATXR3     A D G Y D L V V V D A M H M A N Y A S R I C H S C R P N C E A K V T A V - D G H
hMLL1     - - - - - - E V V D A T M H G N A A R F I N H S C E P N C Y S R V I N I - D G Q
hMLL2     - - - - - - D V V D A T M H G N A A R F I N H S C E P N C F S R V I H V - E G Q
hMLL3     - - - - - - H V I D A T L T G G P A R Y I N H S C A P N C V A E V V T F - E R G
hMLL4     - - - - - - H V I D A T L T G G P A R Y I N H S C A P N C V A E V V T F - D K E
hSET1A    - - - - - - T I I D A T K C G N L A R F I N H C C T P N C Y A K V I T I - E S Q
hSET1B    - - - - - - T I I D A T K C G N F A R F I N H S C N P N C Y A K V I T V - E S Q
yeastSET1 - - - - - - T V I D A T K K G G I A R F I N H C C D P N C T A K I I K V - G G R
```

```
ATX1      E - H I I I F A K R H I P K W E E L T Y D Y R
ATX2      E - H I I I F A K R D V A K W E E L T Y D Y R
ATX3      D N R I V L I A K T N V A A G E E L T Y D Y L
ATX4      S - R I V L I A K A N V A V G E E L T Y D Y L
ATX5      S - R I V L I A K T T V A S C E E L T Y D Y L
ATXR7     K - K I F I Y A K R H I D A G E E I S Y N Y K
ATXR3     Y - Q I G I Y S V R A I E Y G E E I T F D Y N
hMLL1     K - H I V I F A M R K I Y R G E E L T Y D Y K
hMLL2     K - H I V I F A L R R I L R G E E L T Y D Y K
hMLL3     H - K I I I S S S R R I Q K G E E L C Y D Y K
hMLL4     D - K I I I I S S R R I P K G E E L T Y D Y Q
hSET1A    K - K I V I Y S K Q P I G V D E E I T Y D Y K
hSET1B    K - K I V I Y S K Q H I N V N E E I T Y D Y K
yeastSET1 R - R I V I Y A L R D I A A S E E L T Y D Y K
                                          *
```

(grey box) H3 recognition and active center
* Phe/Try switch

Supplementary Figure 17 Amino acid alignment of catalytic domain SET in ATXs and their homologs. Y at ‘Y/F-switch’ is typical for SET domains of mono/di-KMTs [50]. Annotations are from ref. 105.

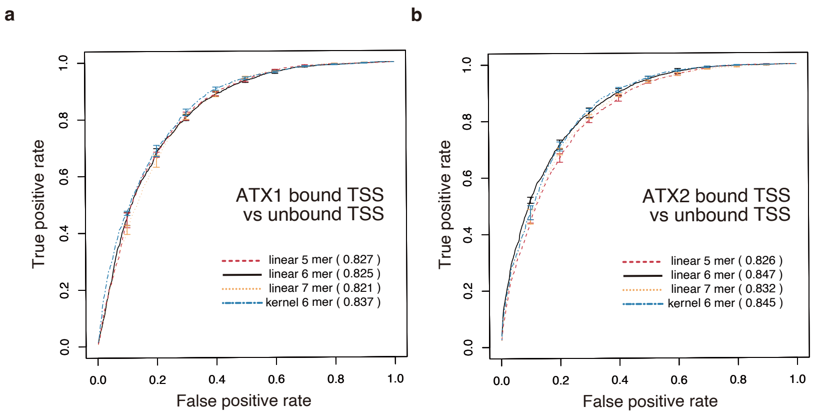

Supplementary Figure 18 Parameter tuning of SVM models.
(a,b) Prediction accuracy of linear SVM models trained with different sizes of k-mers, and kernel SVM with 6-mers. Error bars represent the standard deviation of the 5 repeats of training. The numbers in parenthesis are AUC calculated with test data

References

102. Liu, Y., Koornneef, M. & Soppe, W. J. J. The absence of histone H2B monoubiquitination in the Arabidopsis hub1 (rdo4) mutant reveals a role for chromatin remodeling in seed dormancy. *Plant Cell* **19**, 433–444 (2007).

103. Fleury, D. et al. The Arabidopsis thaliana homolog of yeast BRE1 has a function in cell cycle regulation during early leaf and root growth. *Plant Cell* **19**, 417–432 (2007).

104. Bourbousse, C. et al. Histone H2B monoubiquitination facilitates the rapid modulation of gene expression during Arabidopsis photomorphogenesis. *PLoS Genet.* **8**, e1002825 (2012).

105. Zhang, Y. et al. Evolving catalytic properties of the MLL Family SET Domain. *Struct./Fold. Des*. **23**, 1921–1933 (2015)
