# Supplementary material for: Transcription-coupled and epigenome-encoded mechanisms direct H3K4 methylation
Source: Nat Commun. 2022 Aug 11;13:4521. doi: 10.1038/s41467-022-32165-8 (PMC9372134; doi:10.1038/s41467-022-32165-8)
Supplement: Supplementary file 3 — Description of Additional Supplementary Files [file 41467_2022_32165_MOESM3_ESM.pdf]

Description of Additional Supplementary Files

File Name: Supplementary Data 1
Description: **ChIP-seq replicates confirmed that atx1,atx2,atxr7 having additional impact for H3K4me1.** Summary of the numbers of regions where modifications are significantly reduced compared to WT, detected by MACS2. This table covers five independent (=grown, sampled, and immunoprecipitated on a different day) ChIP-seq datasets; single mutants, double mutants, triple mutants rep.1 and rep.2, and atx1,2,atxr7 replicates.

File Name: Supplementary Data 2
Description: **Summary of spike-in analysis.**

File Name: Supplementary Data 3
Description**: Number of ATX(R) peaks detected by MACS2.** Peaks were called with two settings; the default and more relaxed option (-q 0.3 -- nomodel). The latter was used for Fig. 2d and Supplementary Fig. 5c.

File Name: Supplementary Data 4
Description: **Sources and tissue-of-origin information about public datasets used for Arabidopsis random forest analysis.**

File Name: Supplementary Data 5
Description: **List of all 6-mers and their weights in ATX1 and ATX2 models.** Weights are averaged from 5 cross-validation models. 'ATX1_model' corresponds to a model in Fig. 4, and 'ATX1_model_replicate' corresponds to models based on biological replicate datasets shown in Supplementary Fig. 9.

File Name: Supplementary Data 6
Description: **List of 6-mers in lSVM analyses for ATX and ATX2 and their annotation.** List of highly weighted and unweighted 6-mers, shown in Fig. 4e,f, Supplementary Fig. 10a,b,c, and Supplementary Fig. 11, as well as these 6-mer's best TOMTOM hits that meet the criteria, q-value < 0.1.

File Name: Supplementary Data 7
Description: **Sources of reanalyzed data in mESC used for M. musculus random forest analysis.**
