# Supplementary material for: Transcription-coupled and epigenome-encoded mechanisms direct H3K4 methylation
Source: Nat Commun. 2022 Aug 11;13:4521. doi: 10.1038/s41467-022-32165-8 (PMC9372134; doi:10.1038/s41467-022-32165-8)
Supplement: Supplementary file 11 — Reporting Summary [file 41467_2022_32165_MOESM11_ESM.pdf]

nature portfolio

Corresponding author(s): Satoyo Oya, Soichi Inagaki, Tetsuji Kakutani

Last updated by author(s): 2022/07/15

Reporting Summary

Nature Portfolio wishes to improve the reproducibility of the work that we publish. This form provides structure for consistency and transparency in reporting. For further information on Nature Portfolio policies, see our Editorial Policies and the Editorial Policy Checklist.

Statistics

For all statistical analyses, confirm that the following items are present in the figure legend, table legend, main text, or Methods section.

n/a	Confirmed	
☐	☒	The exact sample size (n) for each experimental group/condition, given as a discrete number and unit of measurement
☐	☒	A statement on whether measurements were taken from distinct samples or whether the same sample was measured repeatedly
☐	☒	The statistical test(s) used AND whether they are one- or two-sided<br>*Only common tests should be described solely by name; describe more complex techniques in the Methods section.*
☐	☒	A description of all covariates tested
☐	☒	A description of any assumptions or corrections, such as tests of normality and adjustment for multiple comparisons
☐	☒	A full description of the statistical parameters including central tendency (e.g. means) or other basic estimates (e.g. regression coefficient) AND variation (e.g. standard deviation) or associated estimates of uncertainty (e.g. confidence intervals)
☐	☒	For null hypothesis testing, the test statistic (e.g. F, t, r) with confidence intervals, effect sizes, degrees of freedom and P value noted<br>*Give P values as exact values whenever suitable.*
☒	☐	For Bayesian analysis, information on the choice of priors and Markov chain Monte Carlo settings
☒	☐	For hierarchical and complex designs, identification of the appropriate level for tests and full reporting of outcomes
☐	☒	Estimates of effect sizes (e.g. Cohen's d, Pearson's r), indicating how they were calculated

Our web collection on statistics for biologists contains articles on many of the points above.

Software and code

Policy information about availability of computer code

Data collection	N/A
Data analysis	bowtie2:2.3.3.1<br>STAR:2.5.3<br>samtools:1.8--4<br>bedtools:2.26.0<br>trimmomatic:0.36--5<br>MACS2:2.1.1<br>IGV:2.5.0<br>ngsplot2.47.1<br>R3.6<br>R package: ROCR1.1<br>R package: viridis0.6.2<br>R package: randomForest4.6-14<br>R package: Biostrings2.54.0<br>R package: qgraph1.9<br>python3<br>Python library: pandas0.25.1<br>Python library: scikit-learn0.21.2<br>Python library: biopython1.74

For manuscripts utilizing custom algorithms or software that are central to the research but not yet described in published literature, software must be made available to editors and reviewers. We strongly encourage code deposition in a community repository (e.g. GitHub). See the Nature Portfolio guidelines for submitting code & software for further information.

Data

Policy information about availability of data

All manuscripts must include a data availability statement. This statement should provide the following information, where applicable:

- Accession codes, unique identifiers, or web links for publicly available datasets
- A description of any restrictions on data availability
- For clinical datasets or third party data, please ensure that the statement adheres to our policy

The high-throughput sequencing data generated in this study are available in NCBI under the accession number PRJNA732996.

Field-specific reporting

Please select the one below that is the best fit for your research. If you are not sure, read the appropriate sections before making your selection.

☒ Life sciences ☐ Behavioural & social sciences ☐ Ecological, evolutionary & environmental sciences

For a reference copy of the document with all sections, see nature.com/documents/nr-reporting-summary-flat.pdf

Life sciences study design

All studies must disclose on these points even when the disclosure is negative.

Sample size	Sample size (biologically independent replicates) is based on field convention for most of experiments; triplicate for RNA-seq (https://genomebiology.biomedcentral.com/articles/10.1186/s13059-016-0881-8#Abs1) and duplicate for ChIP-seq (https://genome.cshlp.org/content/genome/22/9/1813.full.html).
Data exclusions	Scatter plots of NGS-seq signals focus on protein-coding genes, while other transcribed elements such as transposons and tRNA genes are excluded, as stated in legends and within the figure. This is because the focus of this study is regulation over protein-coding genes and not the other genomic elements that are known to be regulated in distinct mechanisms from protein-coding genes.<br>Peaks called within the centromeres are removed, because the centromeres, being highly repetitive and relatively unstable, sometimes attract a large number of artifact peaks. The removed regions here are posted as bedfile in https://github.com/Satoyo08/Arabidopsis_H3K4me1/data/refs/centromere.bed<br>In the contour plot (Figure.5) genes for which no transcription was detected were excluded as stated in the Method. This is because the zero data points do not contribute to capturing the correlational properties.
Replication	All data except for the supplemental H3K4me0 ChIP-seq (Supplementary Figure 1b), WT-ashh2 H3K4me1 ChIP-seq pairs (Supplementary Fig. 6b), and some in-house generated features used in randomforest are based on multiple biologically independent replicates. The number of replicates for each experiment was stated in the Methods, legend or within figure of the manuscript. At least Triplicate for mRNA-seq and duplicate for ChIP-seq.
Randomization	For all experiments, plants were grown on multiple petridishes, which were placed randomly in the growth chamber. In order not to bias the sampling, the researchers conducting sampling were not aware of sample's genotypes.
Blinding	During the process of all experiments, the researchers conducting experiments were not aware of sample's genotypes. For processing sequence data and downstream analysis, genotypes were not blinded because it is impractical. However, raw sequence data codes are publicly available, so that reproducibility of the analyses can be tested.

Reporting for specific materials, systems and methods

We require information from authors about some types of materials, experimental systems and methods used in many studies. Here, indicate whether each material, system or method listed is relevant to your study. If you are not sure if a list item applies to your research, read the appropriate section before selecting a response.

Materials & experimental systems

n/a	Involved in the study	
☐	☒	Antibodies
☒	☐	Eukaryotic cell lines
☒	☐	Palaeontology and archaeology
☒	☐	Animals and other organisms
☒	☐	Human research participants
☒	☐	Clinical data
☒	☐	Dual use research of concern

Methods

n/a	Involved in the study	
☐	☒	ChIP-seq
☒	☐	Flow cytometry
☒	☐	MRI-based neuroimaging

Antibodies

Antibodies used	As stated in the manuscript, anti-H3 (ab1791; Abcam), H3K4me1 (ab8895; Abcam), H3K4me2 (ab32356; Abcam), H3K4me3 (ab8580; Abcam), H3K4me0 (MABI0301, MBL), H3K36me2 (MABI0332; MBL), H3K36me3 (MABI0333; MBL), H3K27me3 (MABI0323; MBL),H2Bub (MM-0029; Medimabs), H4K16ac (07-329; Millipore), RNAP2 total CTD (MABI0601; MBL), RNAP2 phospho S2 (MABI0602; MBL), RNAP2 phospho S5 (MABI0603; MBL), FLAG (F1804; SIGMA), H4 (raised and gifted by Dr. Akihisa Osakabe), Anti-Rabbit HRP (NA934, cytiva).
Validation	The antibodies are not validated specifically for use in Arabidopsis, but all the antibodies are validated for use in ChIP-seq by suppliers, and extensively used in literatures for ChIP-seq and other purposes in Arabidopsis and other species. We validated each antibody by conducting ChIP-seq and comparing the result with previously reported ChIP-seq results of same protein or modification. We validated the specificity of anti-FLAG by comparing ChIP-seq results of transgenic plants expressing FLAG-tagged protein to non-transgenic wild type plants. Although some vendors sell the anti-H3K4me0 antibody as anti-H3,this antibody is specific to unmodified H3K4 as validated in (Kimura, H et al., 2008. Cell Struct. Funct.).

ChIP-seq

Data deposition

☒ Confirm that both raw and final processed data have been deposited in a public database such as GEO.

☐ Confirm that you have deposited or provided access to graph files (e.g. BED files) for the called peaks.

Data access links
May remain private before publication.

https://www.ncbi.nlm.nih.gov/bioproject/PRJNA732996/

Files in database submission

title
ChIP-seq of Arabidopsis thaliana: seedling H3K4me1_WT
ChIP-seq of Arabidopsis thaliana: seedling H3K4me1_atx1
ChIP-seq of Arabidopsis thaliana: seedling H3K4me1_atx2
ChIP-seq of Arabidopsis thaliana: seedling H3K4me1_atx3
ChIP-seq of Arabidopsis thaliana: seedling H3K4me1_atx4
ChIP-seq of Arabidopsis thaliana: seedling H3K4me1_atx5
ChIP-seq of Arabidopsis thaliana: seedling H3K4me1_atxr7
ChIP-seq of Arabidopsis thaliana: seedling H3K4me1_WT_2
ChIP-seq of Arabidopsis thaliana: seedling H3K4me1_atx1/atx2
ChIP-seq of Arabidopsis thaliana: seedling H3K4me1_atx1/atxr7
ChIP-seq of Arabidopsis thaliana: seedling H3K4me1_atx2/atxr7
ChIP-seq of Arabidopsis thaliana: seedling H3K4me2_WT_2
ChIP-seq of Arabidopsis thaliana: seedling H3K4me2_atx1/atx2
ChIP-seq of Arabidopsis thaliana: seedling H3K4me2_atx1/atxr7
ChIP-seq of Arabidopsis thaliana: seedling H3K4me2_atx2/atxr7
ChIP-seq of Arabidopsis thaliana: seedling H3K4me3_WT_2
ChIP-seq of Arabidopsis thaliana: seedling H3K4me3_atx1/atx2
ChIP-seq of Arabidopsis thaliana: seedling H3K4me3_atx1/atxr7
ChIP-seq of Arabidopsis thaliana: seedling H3K4me3_atx2/atxr7
ChIP-seq of Arabidopsis thaliana: seedling H3K4me1_WT_3
ChIP-seq of Arabidopsis thaliana: seedling H3K4me1_atx1/2/r7
ChIP-seq of Arabidopsis thaliana: seedling H3K4me1_atx3/4/5
ChIP-seq of Arabidopsis thaliana: seedling H3K4me1_atx3-1/4/5
ChIP-seq of Arabidopsis thaliana: seedling H3K4me1_atxr3
ChIP-seq of Arabidopsis thaliana: seedling H3K4me2_WT_3
ChIP-seq of Arabidopsis thaliana: seedling H3K4me2_atx1/2/r7
ChIP-seq of Arabidopsis thaliana: seedling H3K4me2_atx3/4/5
ChIP-seq of Arabidopsis thaliana: seedling H3K4me2_atx3-1/4/5
ChIP-seq of Arabidopsis thaliana: seedling H3K4me2_atxr3
ChIP-seq of Arabidopsis thaliana: seedling H3K4me3_WT_3
ChIP-seq of Arabidopsis thaliana: seedling H3K4me3_atx1/2/r7
ChIP-seq of Arabidopsis thaliana: seedling H3K4me3_atx3/4/5
ChIP-seq of Arabidopsis thaliana: seedling H3K4me3_atx3-1/4/5
ChIP-seq of Arabidopsis thaliana: seedling H3K4me3_atxr3
ChIP-seq of Arabidopsis thaliana: seedling H3K36me3_WT
ChIP-seq of Arabidopsis thaliana: seedling H3K36me3_atx1/2/r7
ChIP-seq of Arabidopsis thaliana: seedling H3K36me3_WT_for_ashh2
ChIP-seq of Arabidopsis thaliana: seedling H3K36me3_ashh2
ChIP-seq of Arabidopsis thaliana: seedling H3K36me3_WT_for_ashh34r3
ChIP-seq of Arabidopsis thaliana: seedling H3K36me3_ashh3
ChIP-seq of Arabidopsis thaliana: seedling H3K36me3_ashh4
ChIP-seq of Arabidopsis thaliana: seedling H3K36me3_ashr3
ChIP-seq of Arabidopsis thaliana: seedling H3K36me3_WT_for_ashh1
ChIP-seq of Arabidopsis thaliana: seedling H3K36me3_ashh1
ChIP-seq of Arabidopsis thaliana: seedling RNAP2_WT
ChIP-seq of Arabidopsis thaliana: seedling RNAP2_Ser2P_WT

ChIP-seq of Arabidopsis thaliana: seedling RNAP2_Ser5P_WT
ChIP-seq of Arabidopsis thaliana: seedling Negative_control_for_ATX(R)s_rep1
ChIP-seq of Arabidopsis thaliana: seedling ATX1_rep1
ChIP-seq of Arabidopsis thaliana: seedling ATX2_rep1
ChIP-seq of Arabidopsis thaliana: seedling ATXR7_rep1
ChIP-seq of Arabidopsis thaliana: seedling Negative_control_for_ATXs_rep2
ChIP-seq of Arabidopsis thaliana: seedling ATX1_rep2
ChIP-seq of Arabidopsis thaliana: seedling ATX2_rep2
ChIP-seq of Arabidopsis thaliana: seedling Negative_control_for_ATXR7_rep2
ChIP-seq of Arabidopsis thaliana: seedling ATXR7_rep2
ChIP-seq of Arabidopsis thaliana: seedling H3K36me2
ChIP-seq of Arabidopsis thaliana: seedling H3K27me3
ChIP-seq of Arabidopsis thaliana: seedling H3K4me0_WT
ChIP-seq of Arabidopsis thaliana: seedling H3K4me0_atx1/2/r7
ChIP-seq of Arabidopsis thaliana: seedling H3_WT
ChIP-seq of Arabidopsis thaliana: seedling H3_atx1
ChIP-seq of Arabidopsis thaliana: seedling H3_atx2
ChIP-seq of Arabidopsis thaliana: seedling H3_atx3
ChIP-seq of Arabidopsis thaliana: seedling H3_atx4
ChIP-seq of Arabidopsis thaliana: seedling H3_atx5
ChIP-seq of Arabidopsis thaliana: seedling H3_atxr7
ChIP-seq of Arabidopsis thaliana: seedling H3_WT_2
ChIP-seq of Arabidopsis thaliana: seedling H3_atx1/atx2
ChIP-seq of Arabidopsis thaliana: seedling H3_atx1/atxr7
ChIP-seq of Arabidopsis thaliana: seedling H3_atx2/atxr7
ChIP-seq of Arabidopsis thaliana: seedling H3_WT_3
ChIP-seq of Arabidopsis thaliana: seedling H3_atx1/2/r7
ChIP-seq of Arabidopsis thaliana: seedling H3_atx3/4/5
ChIP-seq of Arabidopsis thaliana: seedling H3_atx3-1/4/5
ChIP-seq of Arabidopsis thaliana: seedling H3_atxr3
ChIP-seq of Arabidopsis thaliana: seedling H3K4me1_WT_for_hub_rep1
ChIP-seq of Arabidopsis thaliana: seedling H3K4me1_hub1_rep1
ChIP-seq of Arabidopsis thaliana: seedling H3K4me1_hub2_rep1
ChIP-seq of Arabidopsis thaliana: seedling H3K4me1_WT_for_hub_rep2
ChIP-seq of Arabidopsis thaliana: seedling H3K4me1_hub1_rep2
ChIP-seq of Arabidopsis thaliana: seedling H3K4me1_hub2_rep2
ChIP-seq of Arabidopsis thaliana: seedling H3_WT_for_hubK4me1_ashhK36me3_atx12r7K36me3_rep2
ChIP-seq of Arabidopsis thaliana: seedling H3_hub1_rep2
ChIP-seq of Arabidopsis thaliana: seedling H3_hub2_rep2
ChIP-seq of Arabidopsis thaliana: seedling H2Bub_WT_rep1
ChIP-seq of Arabidopsis thaliana: seedling H2Bub_atx1/2/r7_rep1
ChIP-seq of Arabidopsis thaliana: seedling H2Bub_WT_rep2
ChIP-seq of Arabidopsis thaliana: seedling H2Bub_atx1/2/r7_rep2
ChIP-seq of Arabidopsis thaliana: seedling H4K16ac_WT_rep1
ChIP-seq of Arabidopsis thaliana: seedling H4K16ac_atx1/2/r7_rep1
ChIP-seq of Arabidopsis thaliana: seedling H4K16ac_WT_rep2
ChIP-seq of Arabidopsis thaliana: seedling H4K16ac_atx1/2/r7_rep2
ChIP-seq of Arabidopsis thaliana: seedling H3K36me3_WT_for_ashhs_atx1/2/r7_rep2
ChIP-seq of Arabidopsis thaliana: seedling H3K36me3_ashh1_rep2
ChIP-seq of Arabidopsis thaliana: seedling H3K36me3_ashh2_rep2
ChIP-seq of Arabidopsis thaliana: seedling H3K36me3_ashh3_rep2
ChIP-seq of Arabidopsis thaliana: seedling H3K36me3_ashh4_rep2
ChIP-seq of Arabidopsis thaliana: seedling H3K36me3_ashr3_rep2
ChIP-seq of Arabidopsis thaliana: seedling H3K36me3_atx1/2/r7_rep2
ChIP-seq of Arabidopsis thaliana: seedling H3_ashh1_rep2
ChIP-seq of Arabidopsis thaliana: seedling H3_ashh2_rep2
ChIP-seq of Arabidopsis thaliana: seedling H3_ashh3_rep2
ChIP-seq of Arabidopsis thaliana: seedling H3_ashh4_rep2
ChIP-seq of Arabidopsis thaliana: seedling H3_ashr3_rep2
ChIP-seq of Arabidopsis thaliana: seedling ATX2_HUB2_rep1
ChIP-seq of Arabidopsis thaliana: seedling ATX2_hub2_rep1
ChIP-seq of Arabidopsis thaliana: seedling ATX2_HUB2_rep2
ChIP-seq of Arabidopsis thaliana: seedling ATX2_hub2_rep2
ChIP-seq of Arabidopsis thaliana: seedling H3K4me1_WT_spike_in
ChIP-seq of Arabidopsis thaliana: seedling H3K4me1_atx1_spike_in
ChIP-seq of Arabidopsis thaliana: seedling H3K4me1_atx2_spike_in
ChIP-seq of Arabidopsis thaliana: seedling H3K4me1_atxr7_spike_in
ChIP-seq of Arabidopsis thaliana: seedling H3K4me1_atx1/atx2_spike_in
ChIP-seq of Arabidopsis thaliana: seedling H3K4me1_atx1/atxr7_spike_in
ChIP-seq of Arabidopsis thaliana: seedling H3K4me1_atx2/atxr7_spike_in
ChIP-seq of Arabidopsis thaliana: seedling H3K4me1_atx1/atx2/atxr7_spike_in
ChIP-seq of Arabidopsis thaliana: seedling H3_WT_spike_in
ChIP-seq of Arabidopsis thaliana: seedling H3_atx1_spike_in
ChIP-seq of Arabidopsis thaliana: seedling H3_atx2_spike_in
ChIP-seq of Arabidopsis thaliana: seedling H3_atxr7_spike_in
ChIP-seq of Arabidopsis thaliana: seedling H3_atx1/atx2_spike_in

ChIP-seq of Arabidopsis thaliana: seedling H3_atx1/atxr7_spike_in
ChIP-seq of Arabidopsis thaliana: seedling H3_atx2/atxr7_spike_in
ChIP-seq of Arabidopsis thaliana: seedling H3_atx1/atx2/atxr7_spike_in
ChIP-seq of Arabidopsis thaliana: seedling H3K4me1_WT_4
ChIP-seq of Arabidopsis thaliana: seedling H3K4me1_atx1/2/r7_4
ChIP-seq of Arabidopsis thaliana: seedling H3K4me1_atx3/4/5_4
ChIP-seq of Arabidopsis thaliana: seedling H3K4me1_atxr3_4
ChIP-seq of Arabidopsis thaliana: seedling H3K4me2_WT_4
ChIP-seq of Arabidopsis thaliana: seedling H3K4me2_atx1/2/r7_4
ChIP-seq of Arabidopsis thaliana: seedling H3K4me2_atx3/4/5_4
ChIP-seq of Arabidopsis thaliana: seedling H3K4me2_atxr3_4
ChIP-seq of Arabidopsis thaliana: seedling H3K4me3_WT_4
ChIP-seq of Arabidopsis thaliana: seedling H3K4me3_atx1/2/r7_4
ChIP-seq of Arabidopsis thaliana: seedling H3K4me3_atx3/4/5_4
ChIP-seq of Arabidopsis thaliana: seedling H3K4me3_atxr3_4
ChIP-seq of Arabidopsis thaliana: seedling H3_WT_4
ChIP-seq of Arabidopsis thaliana: seedling H3_atx1/2/r7_4
ChIP-seq of Arabidopsis thaliana: seedling H3_atx3/4/5_4
ChIP-seq of Arabidopsis thaliana: seedling H3_atxr3_4
ChIP-seq of Arabidopsis thaliana: seedling H3K4me1_WT_for_ashh2
ChIP-seq of Arabidopsis thaliana: seedling H3K4me1_ashh2

Genome browser session
(e.g. UCSC)

Provide a link to an anonymized genome browser session for "Initial submission" and "Revised version" documents only, to enable peer review. Write "no longer applicable" for "Final submission" documents.

Methodology

Replicates

All ChIP-seq experiments were conducted with 2 biological replicates, except for one pair of supplemental result (H3K4me0 ChIP-seq, Used only in Supplementary Fig. 1b) and in-house generated feature data used for random forest (H3K27me3, H3K36me2 etc), which were validated against published datasets.

Sequencing depth

title #pair/single #read length # reads processed: # reads with at least one reported alignment:
ChIP-seq of Arabidopsis thaliana: seedling H3K4me1_WT single 50 bp 17760699 15658338
ChIP-seq of Arabidopsis thaliana: seedling H3K4me1_atx1 single 50 bp 18320643 12075622
ChIP-seq of Arabidopsis thaliana: seedling H3K4me1_atx2 single 50 bp 9692518 6555752
ChIP-seq of Arabidopsis thaliana: seedling H3K4me1_atx3 single 50 bp 11270052 7475471
ChIP-seq of Arabidopsis thaliana: seedling H3K4me1_atx4 single 50 bp 18760044 17217011
ChIP-seq of Arabidopsis thaliana: seedling H3K4me1_atx5 single 50 bp 22530325 19892414
ChIP-seq of Arabidopsis thaliana: seedling H3K4me1_atxr7 single 50 bp 9738380 9024365
ChIP-seq of Arabidopsis thaliana: seedling H3K4me1_WT_2 single 50 bp 26627834 23346131
ChIP-seq of Arabidopsis thaliana: seedling H3K4me1_atx1/atx2 single 50 bp 31492251 26581246
ChIP-seq of Arabidopsis thaliana: seedling H3K4me1_atx1/atxr7 single 50 bp 36011321 30501585
ChIP-seq of Arabidopsis thaliana: seedling H3K4me1_atx2/atxr7 single 50 bp 28761373 25124284
ChIP-seq of Arabidopsis thaliana: seedling H3K4me2_WT_2 single 50 bp 18538238 16668710
ChIP-seq of Arabidopsis thaliana: seedling H3K4me2_atx1/atx2 single 50 bp 16814321 14887538
ChIP-seq of Arabidopsis thaliana: seedling H3K4me2_atx1/atxr7 single 50 bp 21702186 18739036
ChIP-seq of Arabidopsis thaliana: seedling H3K4me2_atx2/atxr7 single 50 bp 22507846 20178072
ChIP-seq of Arabidopsis thaliana: seedling H3K4me3_WT_2 single 50 bp 20103517 17894143
ChIP-seq of Arabidopsis thaliana: seedling H3K4me3_atx1/atx2 single 50 bp 20400992 18427950
ChIP-seq of Arabidopsis thaliana: seedling H3K4me3_atx1/atxr7 single 50 bp 19897725 16944106
ChIP-seq of Arabidopsis thaliana: seedling H3K4me3_atx2/atxr7 single 50 bp 22370476 20453119
ChIP-seq of Arabidopsis thaliana: seedling H3K4me1_WT_3 single 50 bp 22074773 19408019
ChIP-seq of Arabidopsis thaliana: seedling H3K4me1_atx1/2/r7 single 50 bp 29069108 25214413
ChIP-seq of Arabidopsis thaliana: seedling H3K4me1_atx3/4/5 single 50 bp 30188277 26024603
ChIP-seq of Arabidopsis thaliana: seedling H3K4me1_atx3-1/4/5 single 50 bp 21581387 19623596
ChIP-seq of Arabidopsis thaliana: seedling H3K4me1_atxr3 single 50 bp 26610839 23544954
ChIP-seq of Arabidopsis thaliana: seedling H3K4me2_WT_3 single 50 bp 14427601 12100846
ChIP-seq of Arabidopsis thaliana: seedling H3K4me2_atx1/2/r7 single 50 bp 18869714 16530029
ChIP-seq of Arabidopsis thaliana: seedling H3K4me2_atx3/4/5 single 50 bp 18936894 16710281
ChIP-seq of Arabidopsis thaliana: seedling H3K4me2_atx3-1/4/5 single 50 bp 12494313 11499649
ChIP-seq of Arabidopsis thaliana: seedling H3K4me2_atxr3 single 50 bp 33526424 30685139
ChIP-seq of Arabidopsis thaliana: seedling H3K4me3_WT_3 single 50 bp 14236122 12325706
ChIP-seq of Arabidopsis thaliana: seedling H3K4me3_atx1/2/r7 single 50 bp 16434992 14098641
ChIP-seq of Arabidopsis thaliana: seedling H3K4me3_atx3/4/5 single 50 bp 17573210 15556475
ChIP-seq of Arabidopsis thaliana: seedling H3K4me3_atx3-1/4/5 single 50 bp 14946431 13632089
ChIP-seq of Arabidopsis thaliana: seedling H3K4me3_atxr3 single 50 bp 18034881 15663641
ChIP-seq of Arabidopsis thaliana: seedling H3K36me3_WT single 50 bp 23838211 21322101
ChIP-seq of Arabidopsis thaliana: seedling H3K36me3_atx1/2/r7 single 50 bp 25079619 21901322
ChIP-seq of Arabidopsis thaliana: seedling H3K36me3_WT_for_ashh2 single 50 bp 40049535 36947896
ChIP-seq of Arabidopsis thaliana: seedling H3K36me3_ashh2 single 50 bp 42394630 9420919
ChIP-seq of Arabidopsis thaliana: seedling H3K36me3_WT_for_ashh34r3 single 50 bp 24359631 21915675
ChIP-seq of Arabidopsis thaliana: seedling H3K36me3_ashh3 single 50 bp 26825064 25052714
ChIP-seq of Arabidopsis thaliana: seedling H3K36me3_ashh4 single 50 bp 28437980 26080426
ChIP-seq of Arabidopsis thaliana: seedling H3K36me3_ashr3 single 50 bp 21323535 19413721
ChIP-seq of Arabidopsis thaliana: seedling H3K36me3_WT_for_ashh1 single 50 bp 16378522

ChIP-seq of Arabidopsis thaliana: seedling H3K36me3_ashh1 single 50 bp 19310669
ChIP-seq of Arabidopsis thaliana: seedling RNAP2_WT single 50 bp 25207211 20737479
ChIP-seq of Arabidopsis thaliana: seedling RNAP2_Ser2P_WT single 50 bp 23206212 19254621
ChIP-seq of Arabidopsis thaliana: seedling RNAP2_Ser5P_WT single 50 bp 24404755 20684758
ChIP-seq of Arabidopsis thaliana: seedling Negative_control_for_ATX(R)s_rep1 single 50 bp 32596690 20942543
ChIP-seq of Arabidopsis thaliana: seedling ATX1_rep1 single 50 bp 41948815 30928804
ChIP-seq of Arabidopsis thaliana: seedling ATX2_rep1 single 50 bp 45881080 38136170
ChIP-seq of Arabidopsis thaliana: seedling ATXR7_rep1 single 50 bp 38116550 31413116
ChIP-seq of Arabidopsis thaliana: seedling Negative_control_for_ATXs_rep2 single 50 bp 17188787 7224982
ChIP-seq of Arabidopsis thaliana: seedling ATX1_rep2 single 50 bp 19735258 10599041
ChIP-seq of Arabidopsis thaliana: seedling ATX2_rep2 single 50 bp 23923463 18057821
ChIP-seq of Arabidopsis thaliana: seedling Negative_control_for_ATXR7_rep2 single 50 bp 16069780 10287180
ChIP-seq of Arabidopsis thaliana: seedling ATXR7_rep2 single 50 bp 15493671 13155327
ChIP-seq of Arabidopsis thaliana: seedling H3K36me2 single 50 bp 24395250 20808938
ChIP-seq of Arabidopsis thaliana: seedling H3K27me3 single 50 bp 24645334 21359639
ChIP-seq of Arabidopsis thaliana: seedling H3K4me0_WT single 50 bp 12209773 5635842
ChIP-seq of Arabidopsis thaliana: seedling H3K4me0_atx1/2/r7 single 50 bp 12006500 5998395
ChIP-seq of Arabidopsis thaliana: seedling H3_WT single 50 bp 11219811 10274228
ChIP-seq of Arabidopsis thaliana: seedling H3_atx1 single 50 bp 19780122 13259187
ChIP-seq of Arabidopsis thaliana: seedling H3_atx2 single 50 bp 24469936 15988292
ChIP-seq of Arabidopsis thaliana: seedling H3_atx3 single 50 bp 13406514 9342586
ChIP-seq of Arabidopsis thaliana: seedling H3_atx4 single 50 bp 24018242 16262987
ChIP-seq of Arabidopsis thaliana: seedling H3_atx5 single 50 bp 21076517 19021454
ChIP-seq of Arabidopsis thaliana: seedling H3_atxr7 single 50 bp 11217912 10366340
ChIP-seq of Arabidopsis thaliana: seedling H3_WT_2 single 50 bp 28215424 18140439
ChIP-seq of Arabidopsis thaliana: seedling H3_atx1/atx2 single 50 bp 27578849 17613587
ChIP-seq of Arabidopsis thaliana: seedling H3_atx1/atxr7 single 50 bp 26543809 16121770
ChIP-seq of Arabidopsis thaliana: seedling H3_atx2/atxr7 single 50 bp 27892978 18975000
ChIP-seq of Arabidopsis thaliana: seedling H3_WT_3 single 50 bp 28264715 18773802
ChIP-seq of Arabidopsis thaliana: seedling H3_atx1/2/r7 single 50 bp 21692680 15009989
ChIP-seq of Arabidopsis thaliana: seedling H3_atx3/4/5 single 50 bp 22144850 14524623
ChIP-seq of Arabidopsis thaliana: seedling H3_atx3-1/4/5 single 50 bp 16890959 10924738
ChIP-seq of Arabidopsis thaliana: seedling H3_atxr3 single 50 bp 25588692 16954459
ChIP-seq of Arabidopsis thaliana: seedling H3K4me1_WT_for_hub_rep1 paired 150 bp 63238646 62952519
ChIP-seq of Arabidopsis thaliana: seedling H3K4me1_hub1_rep1 paired 150 bp 57129940 56852753
ChIP-seq of Arabidopsis thaliana: seedling H3K4me1_hub2_rep1 paired 150 bp 54989014 54758074
ChIP-seq of Arabidopsis thaliana: seedling H3K4me1_WT_for_hub_rep2 paired 150 bp 21403950 19947571
ChIP-seq of Arabidopsis thaliana: seedling H3K4me1_hub1_rep2 paired 150 bp 21809654 20699006
ChIP-seq of Arabidopsis thaliana: seedling H3K4me1_hub2_rep2 paired 150 bp 21587476 18872252
ChIP-seq of Arabidopsis thaliana: seedling H3_WT_for_hubK4me1_ashhK36me3_atx12r7K36me3_rep2 paired 150 bp 23984260 22408709
ChIP-seq of Arabidopsis thaliana: seedling H3_hub1_rep2 paired 150 bp 24450524 23386543
ChIP-seq of Arabidopsis thaliana: seedling H3_hub2_rep2 paired 150 bp 25368480 23820041
ChIP-seq of Arabidopsis thaliana: seedling H2Bub_WT_rep1 paired 150 bp 35115138 16520608
ChIP-seq of Arabidopsis thaliana: seedling H2Bub_atx1/2/r7_rep1 paired 150 bp 31297396 21006377
ChIP-seq of Arabidopsis thaliana: seedling H2Bub_WT_rep2 paired 150 bp 28482728 20084488
ChIP-seq of Arabidopsis thaliana: seedling H2Bub_atx1/2/r7_rep2 paired 150 bp 26804924 17356915
ChIP-seq of Arabidopsis thaliana: seedling H4K16ac_WT_rep1 paired 150 bp 44615412 42361508
ChIP-seq of Arabidopsis thaliana: seedling H4K16ac_atx1/2/r7_rep1 paired 150 bp 50977738 48301869
ChIP-seq of Arabidopsis thaliana: seedling H4K16ac_WT_rep2 paired 150 bp 20403606 16762746
ChIP-seq of Arabidopsis thaliana: seedling H4K16ac_atx1/2/r7_rep2 paired 150 bp 20472204 15689765
ChIP-seq of Arabidopsis thaliana: seedling H3K36me3_WT_for_ashhs_atx1/2/r7_rep2 paired 150 bp 22179488 20425018
ChIP-seq of Arabidopsis thaliana: seedling H3K36me3_ashh1_rep2 paired 150 bp 21514354 20705123
ChIP-seq of Arabidopsis thaliana: seedling H3K36me3_ashh2_rep2 paired 150 bp 18335104 17730236
ChIP-seq of Arabidopsis thaliana: seedling H3K36me3_ashh3_rep2 paired 150 bp 19176826 18511267
ChIP-seq of Arabidopsis thaliana: seedling H3K36me3_ashh4_rep2 paired 150 bp 18479002 17754373
ChIP-seq of Arabidopsis thaliana: seedling H3K36me3_ashr3_rep2 paired 150 bp 27755088 26064705
ChIP-seq of Arabidopsis thaliana: seedling H3K36me3_atx1/2/r7_rep2 paired 150 bp 16811452 15248961
ChIP-seq of Arabidopsis thaliana: seedling H3_ashh1_rep2 paired 150 bp 23183660 21149689
ChIP-seq of Arabidopsis thaliana: seedling H3_ashh2_rep2 paired 150 bp 21295188 19374727
ChIP-seq of Arabidopsis thaliana: seedling H3_ashh3_rep2 paired 150 bp 22965566 22139955
ChIP-seq of Arabidopsis thaliana: seedling H3_ashh4_rep2 paired 150 bp 20634818 19569056
ChIP-seq of Arabidopsis thaliana: seedling H3_ashr3_rep2 paired 150 bp 27018718 24972395
ChIP-seq of Arabidopsis thaliana: seedling ATX2_HUB2_rep1 paired 150 bp 12817508 11996632
ChIP-seq of Arabidopsis thaliana: seedling ATX2_hub2_rep1 paired 150 bp 31061348 28460608
ChIP-seq of Arabidopsis thaliana: seedling ATX2_HUB2_rep2 paired 150 bp 33845004 19988483
ChIP-seq of Arabidopsis thaliana: seedling ATX2_hub2_rep2 paired 150 bp 22350634 20572270
ChIP-seq of Arabidopsis thaliana: seedling H3K4me1_WT_spike_in paired 150 bp 31105318 26711253
ChIP-seq of Arabidopsis thaliana: seedling H3K4me1_atx1_spike_in paired 150 bp 28562542 24565168
ChIP-seq of Arabidopsis thaliana: seedling H3K4me1_atx2_spike_in paired 150 bp 30317590 22876304
ChIP-seq of Arabidopsis thaliana: seedling H3K4me1_atxr7_spike_in paired 150 bp 28907610 24072398
ChIP-seq of Arabidopsis thaliana: seedling H3K4me1_atx1/atx2_spike_in paired 150 bp 31965890 25507738
ChIP-seq of Arabidopsis thaliana: seedling H3K4me1_atx1/atxr7_spike_in paired 150 bp 26822188 24775741
ChIP-seq of Arabidopsis thaliana: seedling H3K4me1_atx2/atxr7_spike_in paired 150 bp 32688976 27500247
ChIP-seq of Arabidopsis thaliana: seedling H3K4me1_atx1/atx2/atxr7_spike_in paired 150 bp 40833852 31173522
ChIP-seq of Arabidopsis thaliana: seedling H3_WT_spike_in paired 150 bp 35611368 31378322

ChIP-seq of Arabidopsis thaliana: seedling H3_atx1_spike_in paired 150 bp 29455752 27329718
ChIP-seq of Arabidopsis thaliana: seedling H3_atx2_spike_in paired 150 bp 25665118 24008405
ChIP-seq of Arabidopsis thaliana: seedling H3_atxr7_spike_in paired 150 bp 32876612 31296762
ChIP-seq of Arabidopsis thaliana: seedling H3_atx1/atx2_spike_in paired 150 bp 24710624 22995531
ChIP-seq of Arabidopsis thaliana: seedling H3_atx1/atxr7_spike_in paired 150 bp 29380732 28598870
ChIP-seq of Arabidopsis thaliana: seedling H3_atx2/atxr7_spike_in paired 150 bp 29548854 27514967
ChIP-seq of Arabidopsis thaliana: seedling H3_atx1/atx2/atxr7_spike_in paired 150 bp 34194922 31885883
ChIP-seq of Arabidopsis thaliana: seedling H3K4me1_WT_4 paired 150 bp 28050272 24526584
ChIP-seq of Arabidopsis thaliana: seedling H3K4me1_atx1/2/r7_4 paired 150 bp 29926844 19117687
ChIP-seq of Arabidopsis thaliana: seedling H3K4me1_atx3/4/5_4 paired 150 bp 21659882 18468221
ChIP-seq of Arabidopsis thaliana: seedling H3K4me1_atxr3_4 paired 150 bp 26109196 21989128
ChIP-seq of Arabidopsis thaliana: seedling H3K4me2_WT_4 paired 150 bp 12151350 10867100
ChIP-seq of Arabidopsis thaliana: seedling H3K4me2_atx1/2/r7_4 paired 150 bp 10570556 8438303
ChIP-seq of Arabidopsis thaliana: seedling H3K4me2_atx3/4/5_4 paired 150 bp 17453610 13821438
ChIP-seq of Arabidopsis thaliana: seedling H3K4me2_atxr3_4 paired 150 bp 10796008 9187892
ChIP-seq of Arabidopsis thaliana: seedling H3K4me3_WT_4 paired 150 bp 13327778 10524449
ChIP-seq of Arabidopsis thaliana: seedling H3K4me3_atx1/2/r7_4 paired 150 bp 12051598 9374062
ChIP-seq of Arabidopsis thaliana: seedling H3K4me3_atx3/4/5_4 paired 150 bp 11992600 9293764
ChIP-seq of Arabidopsis thaliana: seedling H3K4me3_atxr3_4 paired 150 bp 15853870 10598950
ChIP-seq of Arabidopsis thaliana: seedling H3_WT_4 paired 150 bp 18752936 17954692
ChIP-seq of Arabidopsis thaliana: seedling H3_atx1/2/r7_4 paired 150 bp 21218320 19239655
ChIP-seq of Arabidopsis thaliana: seedling H3_atx3/4/5_4 paired 150 bp 21542808 18638854
ChIP-seq of Arabidopsis thaliana: seedling H3_atxr3_4 paired 150 bp 20291266 16809696
ChIP-seq of Arabidopsis thaliana: seedling H3K4me1_WT_for_ashh2 single 50 bp 26879160 24217033
ChIP-seq of Arabidopsis thaliana: seedling H3K4me1_ashh2 single 50 bp 20195428 18172424

Antibodies

anti-H3 (ab1791; Abcam), H3K4me1 (ab8895; Abcam), H3K4me2 (ab32356; Abcam), H3K4me3 (ab8580; Abcam), H3K4me0 (MABI0301, MBL), H3K36me2 (MABI0332; MBL), H3K36me3 (MABI0333; MBL), H3K27me3 (MABI0323; MBL), H2Bub (MM-0029; Medimabs), H4K16ac (07-329; Millipore), RNAP2 total CTD (MABI0601; MBL), RNAP2 phospho S2 (MABI0602; MBL), RNAP2 phospho S5 (MABI0603; MBL), FLAG (F1804; SIGMA)

Peak calling parameters

As stated in the method section, all peaks were called with MACS2. Differentially modified regions were identified by comparing mutant to WT using MACS2 with option -g 1.3e8. Enrichment peaks of H3K4 methyltransferases were identified by comparing against negative control (ChIP-seq done with FLAG antibody in non-transgenic WT) with two settings; the default(-g 1.3e8) and more relaxed option (-q 0.3 --nomodel -g 1.3e8). Both counts are provided as Supplementary Data.3 and the latter was used for Fig.2 d and Supplementary Fig. 5c.

Data quality

Data quality estimation was based on the mapping statistics and the visual inspection in IGV browser, metaplot, and scatterplot comparing replicates.

Software

bowtie2:2.3.3.1
STAR:2.5.3
samtools:1.8--4
bedtools:2.26.0
trimmomatic:0.36--5
MACS2:2.1.1
IGV:2.5.0
ngsplot2.47.1
