# Supplementary material for: Transcription-coupled and epigenome-encoded mechanisms direct H3K4 methylation
Source: Nat Commun. 2022 Aug 11;13:4521. doi: 10.1038/s41467-022-32165-8 (PMC9372134; doi:10.1038/s41467-022-32165-8)
Supplement: Supplementary file 12 — Source Data [file 41467_2022_32165_MOESM12_ESM.zip › source data.pdf]

Source Data

(a-g) Uncropped blots behind Figure 1e **(a-e)** and Supplementary Figure 2a **(f,g)**. **(a-c)**, **(d-e)** and **(f-g)** are respectively the same membrane. **(b)** was acquired with a longer exposure than **(a)**. **(c,e,g)** are chemical luminescence signals in **(b,d,f)** overlayed on membrane view. HMW, high molecular weight; LMW, low molecular weight. **(h)** relative signal of H3K4me1 in *atx1/2/r7* compared to WT. The values behind Supplementary Figure 2a.

a

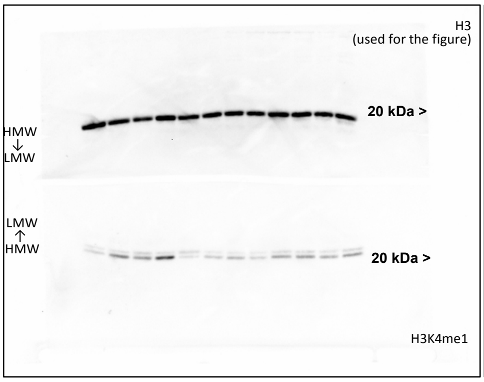

b

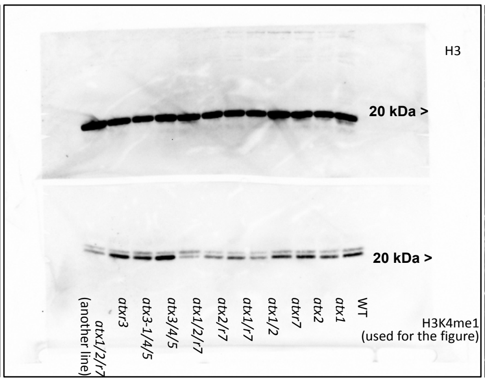

c **overlay of chemical luminecence (b) + membrane view**
cuts mark the molecular ladder markers (Precision Plus Dua)

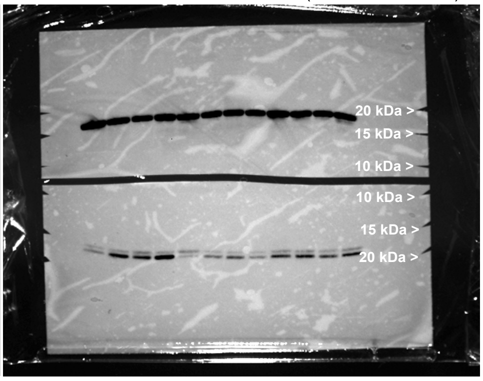

d

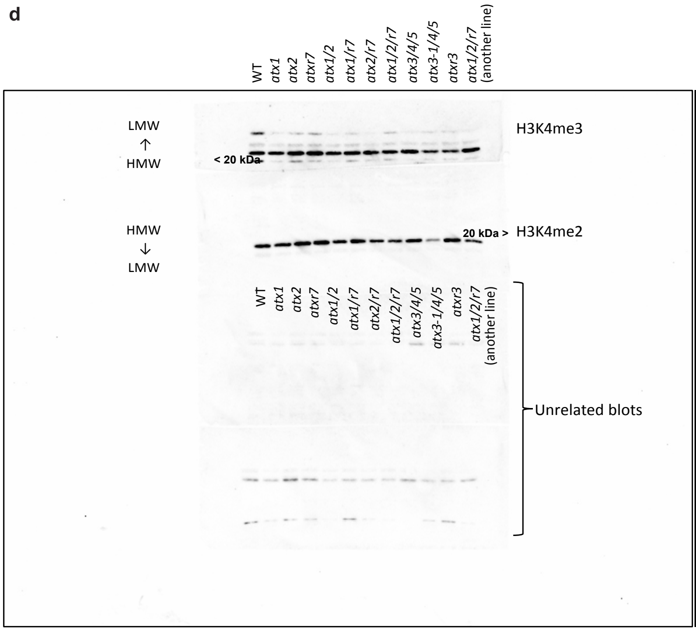

e

overlay of chemical luminecence (d) + membrane view
cuts mark the molecular ladder markers (Precision Plus Dual)

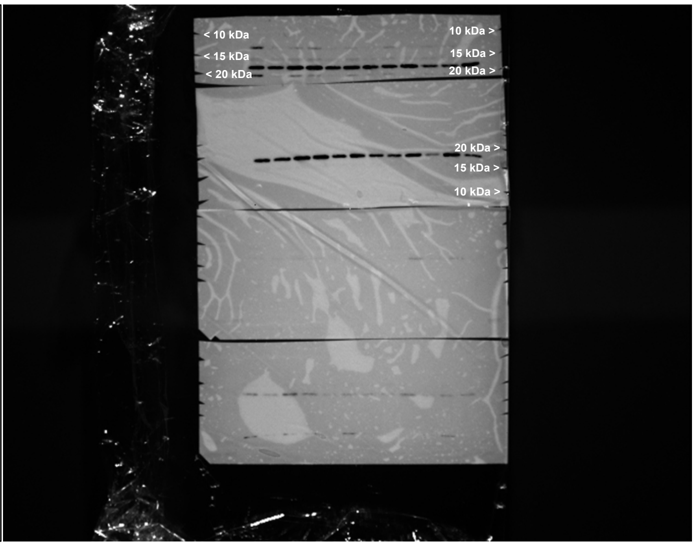

f

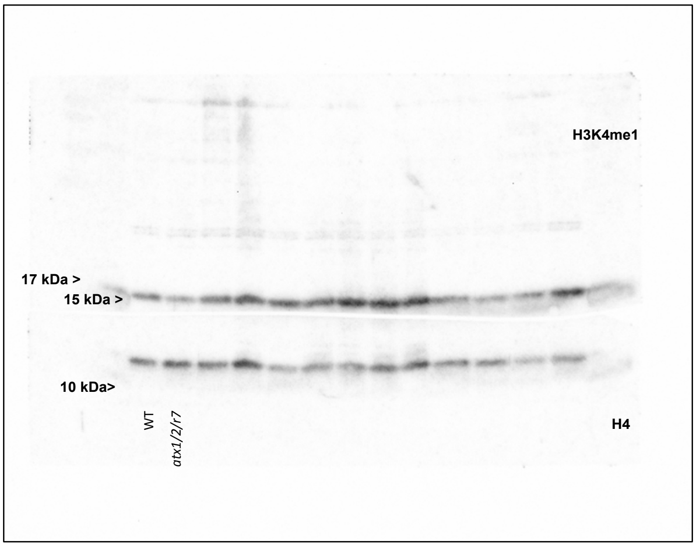

g **overlay of chemical luminecence + membrane view**

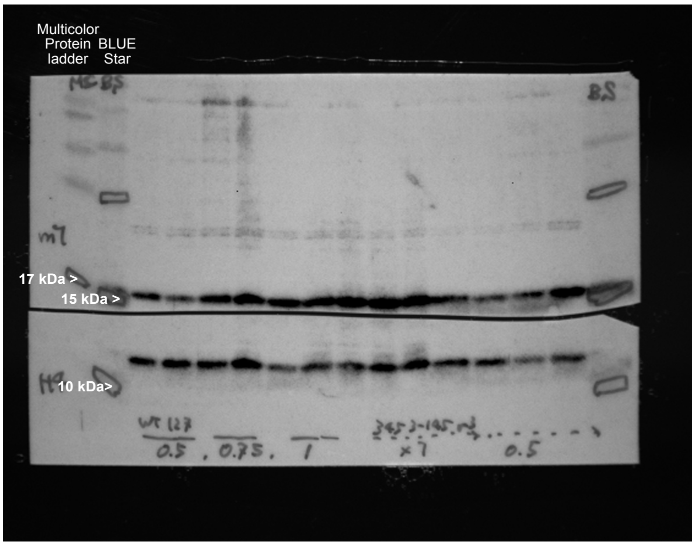

h mutant/WT (H3K4me1/H4,H3)

	rep1	rep2	rep3	rep4	rep5	rep6	rep7	rep8	rep9	rep10	rep11	rep12
**WT**	1	1	1	1	1	1	1	1	1	1	1	1
**atx1/2/r7**	0.5325	0.4701	0.5315	0.9058	0.5212	0.5659	0.8278	0.6915	0.7702	0.8294	NA	0.1727
**atx1/2/r7 (another mutant line)**	NA	NA	NA	NA	NA	NA	NA	0.9590	0.5690	NA	0.3373	0.2227
